# Optimizing Child Nutrition Education With the Foodbot Factory Mobile Health App: Formative Evaluation and Analysis

**DOI:** 10.2196/15534

**Published:** 2020-04-17

**Authors:** Jacqueline Marie Brown, Robert Savaglio, Graham Watson, Allison Kaplansky, Ann LeSage, Janette Hughes, Bill Kapralos, JoAnne Arcand

**Affiliations:** 1 Faculty of Health Sciences University of Ontario Institute of Technology Oshawa, ON Canada; 2 Faculty of Business & Information Technology University of Ontario Institute of Technology Oshawa, ON Canada; 3 Faculty of Community Services Ryerson University Toronto, ON Canada; 4 Faculty of Education University of Ontario Institute of Technology Oshawa, ON Canada

**Keywords:** mHealth, children, child nutrition sciences, mobile apps, health education

## Abstract

**Background:**

Early nutrition interventions to improve food knowledge and skills are critical in enhancing the diet quality of children and reducing the lifelong risk of chronic disease. Despite the rise of mobile health (mHealth) apps and their known effectiveness for improving health behaviors, few evidence-based apps exist to help engage children in learning about nutrition and healthy eating.

**Objective:**

This study aimed to describe the iterative development and user testing of *Foodbot Factory*, a novel nutrition education gamified app for children to use at home or in the classroom and to present data from user testing experiments conducted to evaluate the app.

**Methods:**

An interdisciplinary team of experts in nutrition, education (pedagogy), and game design led to the creation of *Foodbot Factory*. First, a literature review and an environmental scan of the app marketplace were conducted, and stakeholders were consulted to define the key objectives and content of *Foodbot Factory*. Dietitian and teacher stakeholders identified priority age groups and learning objectives. Using a quasi-experimental mixed method design guided by the Iterative Convergent Design for Mobile Health Usability Testing approach, five app user testing sessions were conducted among students (ages 9-12 years). During gameplay, engagement and usability were assessed via direct observations with a semistructured form. After gameplay, qualitative interviews and questionnaires were used to assess user satisfaction, engagement, usability, and knowledge gained.

**Results:**

The environmental scan data revealed that few evidence-based nutrition education apps existed for children. A literature search identified key nutrients of concern for Canadian children and techniques that could be incorporated into the app to engage users in learning. *Foodbot Factory* included characters (2 scientists and Foodbots) who initiate fun and engaging dialogue and challenges (minigames), with storylines incorporating healthy eating messages that align with the established learning objectives. A total of five modules were developed: drinks, vegetables and fruit, grain foods, animal protein foods, and plant protein foods. Seven behavior change techniques and three unique gamified components were integrated into the app. Data from each user testing session were used to inform and optimize the next app iteration. The final user testing session demonstrated that participants agreed that they wanted to play *Foodbot Factory* again (12/17, 71%), that the app is easy to use (12/17, 71%) and fun (14/17, 88%), and that the app goals were clearly presented (15/17, 94%).

**Conclusions:**

*Foodbot Factory* is an engaging and educational mHealth intervention for the Canadian public that is grounded in evidence and developed by an interdisciplinary team of experts. The use of an iterative development approach is a demonstrated method to improve engagement, satisfaction, and usability with each iteration. Children find *Foodbot Factory* to be fun and easy to use, and can engage children in learning about nutrition.

## Introduction

### Background

Poor diet quality is a leading risk factor for chronic disease, such that the risk of morbidity and mortality from a low-quality diet now surpasses the risk associated with tobacco exposure [1]. Globally, the greatest dietary risk factors are high intakes of sodium and low intakes of whole grains, fruits, and vegetables [1]. In Canada, 92% of children consume excess sodium, and on average, children do not consume the recommended servings of whole grains, vegetables, and fruits, findings that are consistent with most other developed countries [2,3]. These dietary risk factors are extremely relevant for children as they require high-quality diets for growth, development, and academic success [4]. Healthy eating behaviors are established during childhood; therefore, it is important to optimize the adoption of these behaviors early in life so that they will be more likely to be sustained into adulthood [5].

Mobile health (mHealth) innovations present novel opportunities to address public health challenges [6]. Systematic reviews and individual studies demonstrate that mHealth interventions improve a variety of dietary and health-related outcomes, such as nutrition knowledge, overall eating patterns, fruit and vegetable intake, intake of nutrients of public health concern (ie, dietary sodium), body weight, blood pressure, and blood cholesterol levels across various populations and age groups, including children and adolescents [7-14]. In addition to health benefits, mHealth interventions are a promising and an innovative way to facilitate nutrition education as children and adolescents increasingly have access to mobile devices at home and school. In Canada, 47% of children aged 0 to 11 years and 80% of adolescents aged 12 to 17 years own a mobile device [15]. However, few evidence-based mHealth nutrition apps for children are publicly available on the app marketplace [16], and there are currently no known apps available to support educators in facilitating nutrition education in the classroom or clinic settings [17,18]. These data point to a significant gap and an opportunity to address an important public health issue in using evidence-based and engaging mHealth nutrition interventions as a way to engage children in learning about healthy eating.

### Objective

The aim of this study was to describe the iterative design, development, and testing of an engaging, evidence-based mHealth nutrition education app, *Foodbot Factory*. The *Foodbot Factory* app was developed as a public health intervention to increase the knowledge and awareness about food and nutrition among healthy children and was designed for use at home and in school. This gamified mHealth app was developed by an interdisciplinary team for children aged 9 to 12 years. The overarching objective of *Foodbot Factory* is to improve food and nutrition knowledge among children.

## Methods

### Study Design

An interdisciplinary team of experts was formed to develop *Foodbot Factory*. This team included experts in nutrition science, behavioral interventions, education, pedagogy and technology, and game design. The team also collaborated with the Office of Nutrition Policy and Promotion at Health Canada who are responsible for improving the health of Canadians through the implementation of nutrition policies and programs.

**Figure 1:**
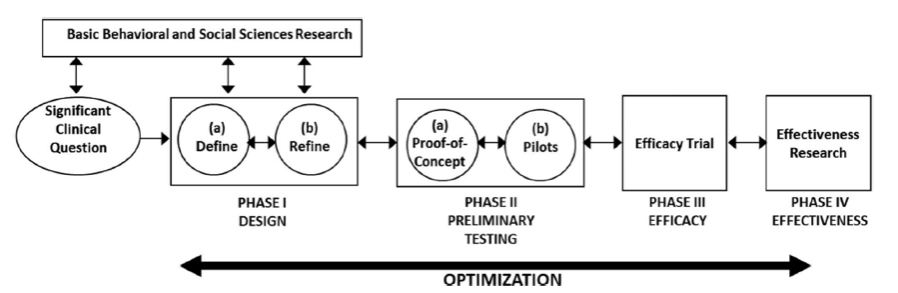
Obesity-Related Behavioral Intervention Trial model.

*Foodbot Factory* development and testing methodologies were guided by the Obesity-Related Behavioral Intervention Trials (ORBIT) model, which is used for developing behavioral interventions for chronic disease prevention and management (Figure 1) [19]. This model is useful for the development of complex interventions as goals and milestones are established for each phase, and developers are encouraged to revisit previous phases to improve the intervention based on new evidence [19]. The ORBIT model also promotes the integration of behavioral and social sciences research by requiring that the design phase is informed by the latest research, ensuring that high-quality evidence is incorporated into the intervention. The ORBIT model was chosen because it emphasizes the process of developing a behavioral intervention as opposed to the actual content, which will vary across disciplines, populations, and scenarios. It was further considered ideal in the development of *Foodbot Factory* as it emphasizes a data-driven iterative approach to optimize successive iterations of the intervention, so that the intervention will effectively achieve its intended objectives. In addition, the ORBIT model is highly complementary to frameworks used by game developers, such as the Design, Play, and Experience framework [20]. This paper has presented the development and evaluation of *Foodbot Factory* over three phases of the ORBIT model—*Discovery, Phase Ia: Define, and Phase Ib: Refine*[19].

### Discovery Phase

During the *Discovery* phase, the central research question was defined, and educational and behavioral strategies to effectively engage children in learning about nutrition were identified [19]. This phase of development involved background research in behavioral sciences, pedagogy, mHealth app development, and nutritional sciences to identify important points for the intervention and effective methods and techniques for delivery. Background research included a systematic literature search of the PubMed database, conducted in October 2017, to identify peer-reviewed research of mHealth nutrition interventions targeted at a child population. A scoping review was also conducted to identify relevant systematic reviews and literature on the dietary habits of Canadian children. An additional environmental scan of existing mHealth resources was conducted to establish a baseline understanding of the learning tools available for nutrition education [16].

### Phase I: Design

The overall goal of *Phase I: Design* was to identify the essential features of the *Foodbot Factory* app, which included identifying the goals and structure and *tinkering* with the nutrition content and educational strategies to refine and improve upon it [19]. This phase required insights on the nutrition education priorities for the target audience, alignment with behavioral theory, and iterative development and testing of *Foodbot Factory*.

#### Phase Ia: Define

For the *Define* phase, three key components were outlined based on the *Discovery Phase* findings: nutrition content, gamification, and behavior change techniques (BCTs) [19]. Key stakeholders were consulted from February to May 2017 to refine the (1) target age group, (2) isolate the highest priority nutrition education issues, and (3) identify the support needed to implement nutrition curricula. Nutrition-focused stakeholders included public health dietitians who were contacted through Dietitians of Canada, Ontario Dietitians in Public Health, and Health Canada. We also consulted with teachers at a local school board. Stakeholders provided input via a Web-based questionnaire that consisted of 22 items on 7-point Likert scale questions. Open-ended questions were also included to capture additional narrative feedback.

Specific learning objectives incorporating the curriculum and public health priorities were defined based on data and feedback from stakeholders. Considering published evidence on best practices for mHealth apps targeted at children, it was decided that learner engagement with the educational content in *Foodbot Factory* would be enhanced by including (1) gamification, which refers to the application of game design elements to engage and motivate users [21], and (2) evidence-based BCTs, which are “the observable, replicable, and irreducible components of an intervention designed to alter or redirect causal processes that regulate behavior” [22]. The *Foodbot Factory* content was defined and redefined by the development team throughout the iterative development process, incorporating the latest scientific evidence and findings from *Phase Ib* user testing results.

#### Phase Ib: Refine

The purpose of the *Refine* phase is to ensure that an intervention is robust and efficient at achieving its intended objectives. Therefore, a quasi-experimental mixed method design was used to structure formal user testing sessions to allow for the examination of the user experience and impact of the intervention on learning. These outcomes were assessed with each iteration of *Foodbot Factory*, a method recommended by the Iterative Convergent Design for Mobile Health Usability Testing approach [19,23]. This mixed method approach provided the development team with rich multi-faceted data on the users’ experience, which were then used to inform subsequent iterations of the intervention. The *Refine* phase included five iterative user testing sessions with the target audience. As various aspects of *Foodbot Factory* were defined in *Phase Ia*, they were tested with users and refined in *Phase Ib.* Throughout the development process, successive iterations of *Foodbot Factory* moved between *Phases Ia* and *Ib* as the app components were optimized for usability and satisfaction to facilitate engagement in learning.

The five iterative user testing sessions were conducted with student participants in grades 4 to 6 (aged 9-12 years) from a local school board and a science and technology summer camp at Ontario Tech University. User testing sessions were approximately 20 to 30 min in duration and began with an explanation of the study procedures, followed by the collection of informed assent from participants and brief instructions on how to use *Foodbot Factory*. The participants tested the app on an Apple iPad (iOS 12) or a Lenovo tablet (Android 8.1.0) for 10 min, followed by interviews and questionnaires for another 10 min. Each student had their own device; however, participants were able to discuss the app with their peers as they played. Parental consent was obtained before user testing sessions through letters of information and consent forms that were sent home with students before the study. No compensation was provided for parents, students, teachers, or camp counsellors.

The user testing sessions assessed satisfaction, engagement, knowledge, and usability, which were chosen to reflect the core features and components necessary for the app to be successful. Satisfaction refers to the user’s subjective experience with *Foodbot Factory* and the appropriateness and suitability of the platform and content for the target audience. Engagement refers to how the target audience interacted with the app, (ie, found the app enjoyable and wanted to play again). Knowledge refers to how well *Foodbot Factory* supported the target audience’s acquisition of information about healthy eating. Usability refers to the technical aspects of the app, (ie, easy to use and navigate and clear presentation of content). These metrics were assessed using direct observations, interviews, and questionnaires.

During gameplay, direct observations were used by the research and development team to evaluate usability and engagement with the app. Direct observations included recording notes and completing an internally developed semistructured form where the rater recorded comments about usability and engagement. At this time, perceived engagement with the app dialogue was also rated on a 5-point Likert scale. After gameplay, one-on-one semistructured interviews were conducted with the student participants. The interviews included five questions to assess user satisfaction and knowledge acquisition, with a focus on obtaining feedback for the next iteration of the app, rather than a formal evaluation of learning. Interview guides were created by the development team. The interviews were not audio- or video-recorded, rather verbatim notes were taken. The verbatim notes were reviewed by the development team to identify priority areas for improvement in the next app iteration. Answers to questions regarding nutrition knowledge were summarized based on student responses and have been presented as frequencies. Finally, participants also completed an 8-item (5-point Likert scale) questionnaire to assess engagement and usability. Questions were adapted from a validated questionnaire for adults as no known validated questionnaire was available to assess game engagement in children [24].

Categorical data are reported as frequencies and percentages, and continuous data are reported as means and standard deviations. Likert scale responses ranged from *strongly disagree* to *strongly agree*. For the analysis, *strongly disagree* and *disagree* were pooled to reflect disagreement with a questionnaire item and *strongly agree* and *agree* were pooled to reflect agreement with a questionnaire item. An unpaired 2-tailed *t* test was used to conduct post hoc analyses that compared user satisfaction between boy and girl participants. SPSS (v 25) was used for the statistical analysis. A *P* value of .05 was considered statistically significant.

Parental consent and child assent were obtained before the user testing sessions. In total, five iterative user testing sessions were conducted. An ethics board approval was obtained from both Ontario Tech University and the Durham Catholic District School Board (file numbers: 14426 and 14879).

## Results

### Discovery

The development process for *Foodbot Factory* began in October 2017, and the research presented in this paper was completed in December 2018. The design process of *Foodbot Factory* was facilitated by an interdisciplinary team who met on a weekly basis to propose ideas, define specific app content, review user testing results, and subsequently refine the app content.

A scoping review identified foods and nutrients of concern for this population and found that the average Canadian child has an inadequate intake of fiber and an excessive intake of saturated fat, sugar, and sodium [25]. Canadian children also consume inadequate amounts of vegetables, fruits, and whole grains and acquire 30% of daily sugar calories from beverages containing free sugars; therefore, the education of these foods and nutrients was considered a priority for the app [3,26].

Existing systematic reviews of mHealth dietary interventions for children and adolescents were identified. These studies indicated that nutrition knowledge acquisition, nutrition-related behaviors, and nutrition-related health outcomes improve with mHealth interventions [7,9], and they highlighted the strengths of gamification as a strategy to implement for child nutrition mHealth apps [27,28]. An additional systematic search, conducted by the *Foodbot Factory* research and development team, focused on identifying other strategies demonstrated to engage children in learning or behavior change through mHealth nutrition apps [10,11,29-35]. Gamification was implemented in two articles and was found to clearly engage children in learning and improving their nutrition knowledge and behaviors and health outcomes [10,31]. In addition, the use of BCTs was also identified as a replicable and potentially effective approach to include in the mHealth app [36]. There was minimal evidence on the most effective BCTs to incorporate for our target audience to achieve the goals of *Foodbot Factory*. Therefore, the development team hypothesized which BCTs would be most effective at facilitating learning among the target audience and evaluated the user experience and learning throughout the iterative development process.

An environmental scan of the app marketplace identified 249 mobile food apps targeted at children [16,37]; however, very few were of high quality, as scored by the Mobile App Rating Scale [38]. Furthermore, gamified apps were more likely to display high-sodium and high-sugar foods and received a higher number of downloads compared with nongamified apps. These results identified gaps in the public app marketplace, where food games are popular with children but are not aligned with the existing dietary recommendations [16,37].

The research conducted in this phase identified research gaps in the development of child nutrition mHealth apps; however, positive findings from the available literature indicated that gamified mHealth apps are a promising avenue to pursue for education. Furthermore, evidence gathered in this phase demonstrated that mHealth nutrition apps are engaging and can be accessed by the majority of the target population because of the ubiquity of mobile technology in Canada [39]. The *Discovery* phase led to the research question: “Can a mHealth nutrition education app improve nutrition knowledge in children aged 9-12?” This question defined the focus of *Foodbot Factory* to improve users’ nutrition knowledge related to foods and nutrients of public health concern [3,25]. With the central research question and strategy identified, the development of *Foodbot Factory* proceeded to *Phase Ia* [19].

### Phase Ia: Define

Dietitian and teacher stakeholders (n=21) reported their perceived priorities in relation to the target audience and learning objectives for the app: 81% (17/21) ranked grades 7 to 8 as the first priority age group and 86% (18/21) ranked grades 5 to 6 as the second priority age group for a nutrition education app. The interdisciplinary app development team decided to target students in grades 4 to 6 (aged 9-12 years) for the app as it was believed this age group would be more interested in a gamified nutrition app compared with older students. Overall, 86% (18/21) of stakeholders agreed that increasing water consumption and decreasing sugary beverage consumption was important for this age group, and 57% (12/21) of them also agreed that understanding the nutritional value of foods was important.

In alignment with stakeholder feedback, the app was designed to meet the 2018 Ontario Grade 4 Health and Physical Education Curriculum expectations that students should “identify the key nutrients (eg, fat, carbohydrates, protein, vitamins, minerals) provided by foods and beverages, and describe their importance for growth, health, learning, and physical performance” [40]. This expectation also aligns with the health curriculum expectations from other Canadian provinces and territories, providing teachers across the country with a mobile tool to aid nutrition education in the classroom [41,42].

The goal of *Foodbot Factory* is to improve nutrition knowledge, a component of food skills that has been identified as a gap in the ability of Canadian youth to make healthy eating choices [43]. Although knowledge is often insufficient to initiate dietary behavior change, it is necessary for individuals to change their behavior and is associated with better diet quality in adolescents [44]. Furthermore, the most recent version of Canada’s Food Guide (CFG), the leading government resource developed by Health Canada for nutrition guidance, has been disseminated entirely through digital platforms [45]. The CFG is a core component of the school curriculum across Canada. However, to date no tailored resources, messaging, or tools have been created for children and teachers, highlighting a significant gap in nutrition education in Canada [40-42]. As our target audience of 9- to 12-year-old moves into adolescence, they will exercise greater autonomy in their eating decisions. It is hypothesized that the nutrition knowledge obtained from *Foodbot Factory* will empower student users to make healthier dietary choices. Therefore, the goal of this app is to provide the baseline knowledge required to make informed healthy eating choices.

#### Nutrition Content

To align *Foodbot Factory* with the latest dietary guidelines, the development team consulted with Health Canada to ensure the app’s content aligned with the messages in the new 2019 CFG recommendations. The nutrition content in *Foodbot Factory* is structured into five modules, corresponding to the CFG food groups and linking to specific learning objectives [45]. Specific learning objectives were developed for the foods and nutrients relevant for each food grouping, as detailed in Table 1 (Multimedia Appendix 1).

**Table 1:** Foodbot Factory modules, nutrients of concern, and learning objectives.

Level (food grouping)	Nutrients of concern included	Module learning objectives
Drinks	Sugar	Recall the best beverage choice for staying hydrated Describe the health effects of different types of beverages Recall different types of sugary drinks
Grain foods	Fiber	Explain the nutritional differences between whole grain and refined grain products Recall the components of the grain kernel and how grains are refined Describe why consuming fiber is important for health
Vegetables and fruits	Fiber, vitamins, and minerals	Recall the amount of vegetables and fruits that should be consumed with a meal Explain why vegetables and fruits are a healthy choice Describe which forms of vegetables and fruit are healthiest to consume (ie, canned, frozen, and juice)
Animal protein foods	Protein, saturated fat, and sodium	Recall that some fats are healthy (unsaturated fats) and unhealthy (saturated fats) Describe the health effects of excess dietary saturated fat and sodium. Explain why processed meats should be consumed less often Describe why fish are a healthy choice
Plant protein foods	Protein and fiber	Describe what foods are plant protein foods and why they are a healthy choice Recall that plant protein foods contain fiber

#### Gamification

**Figure 2:**
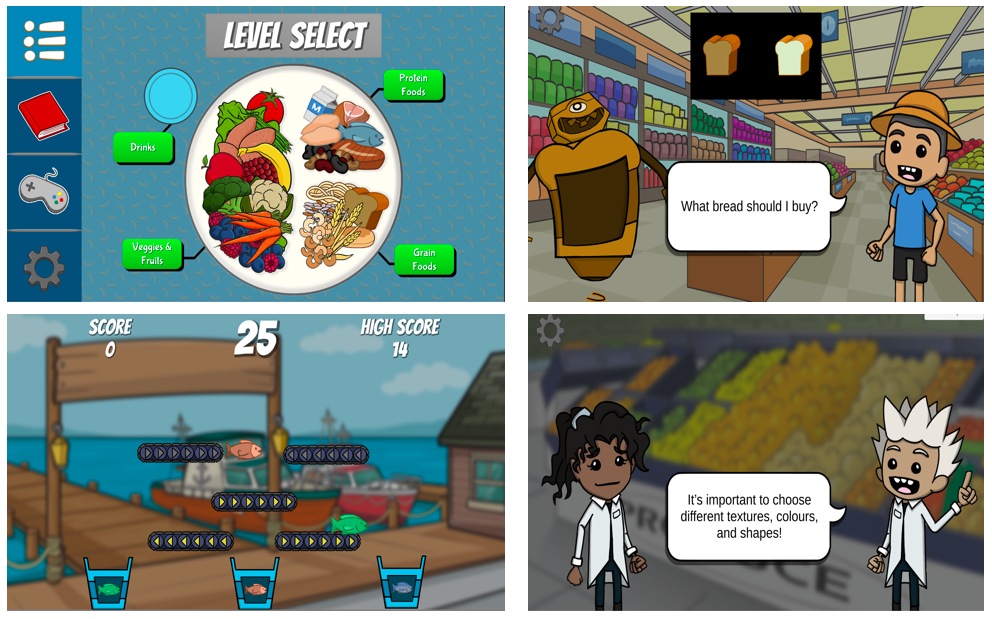
Foodbot Factory screenshots.

The app included three main gamified elements to enhance user interactions, engagement, and motivation to play: quizzes, catching food, and sorting food. *Foodbot Factory* is a story-based app set in a fictional town where residents have nutrition robots (Foodbots) to help them make dietary decisions. The storyline is driven by two nutrition scientists, Robbie and Rebecca, who guide the user on a healthy eating adventure that begins when one of the Foodbots experiences mechanical failure (Figure 2 and Multimedia Appendix 2).

**Figure 3:**
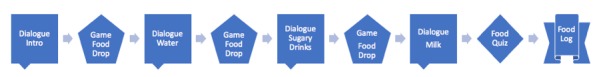
Foodbot Factory game flow diagram for Drinks module.

Each of the five modules has four unique components: dialogue, gameplay, food quizzes, and food logs (Figure 3). Dialogue communicates the nutrition content and allows for game character development, incorporating elements of humor and banter. A total of three modules contain additional interactive dialogue allowing for the users’ choice in the content explored. A Flesch Formula score of 88.9 indicates that the dialogue is easy to read and appropriate for grade 4 readers [46]. Gameplay opportunities are provided intermittently during each module. Gameplay involves collecting or sorting a variety of food items and is varied across the five modules to limit repetition. Food quizzes conclude each module, allowing the users to self-assess their learning. The quizzes allow for self-correction for incorrect responses. The quizzes are interactive, requiring the user to help game characters make healthy eating decisions. The interactive quizzes were designed to simulate real-world dietary choices, which may increase the user’s confidence in making real-life healthy eating decisions. The food log (a compendium of foods introduced in the app) is the final component of each module. The food log provides a platform for the user to enhance and expand their learning and explore food nutrition facts, reinforcing information presented in the module.

*Foodbot Factory* employs multiple gamification characteristics, as defined by the Taxonomy of Gamification Concepts for Health Apps and highlighted in Multimedia Appendix 3 [47]*.* In summary, the app utilizes mediated communication where information is delivered through a guided storyline. Rewards are received within the app, and there is no competition with other players or opportunities for collaboration. As *Foodbot Factory* is educational, the content focuses on learning objectives and does not include goal setting, eg, setting a goal for daily water intake. Users receive positive and negative reinforcement during gameplay. Negative reinforcement occurs during gamified components when users do not sort or catch the correct foods or when a question in the food quiz is answered incorrectly. The persuasive intent of *Foodbot Factory* is to positively change user’s attitudes and knowledge regarding healthy eating.

#### Behavior Change Techniques

BCTs were incorporated into the app to illustrate how the knowledge gained can be applied to actual dietary decisions and increase self-efficacy around these decisions. The main BCT categories integrated throughout *Foodbot Factory* are feedback and monitoring, social support, shaping knowledge, natural consequences, and reward and threat [22]. In total, *Foodbot Factory* utilizes seven BCTs across the game (Table 2) [22]. In each module, users have the opportunity to self-assess their knowledge by helping game characters make dietary decisions. The users are then provided with feedback on the accuracy of those dietary decisions. Users also learn about the natural health consequences that result from consuming certain foods and nutrients. At the end of each module, users are rewarded by unlocking new food items in the food log, where they can review additional nutrition information.

**Table 2:** Behavior change techniques implemented in Foodbot Factory.

Module	Behavior change techniques implemented
Drinks	2.2 Feedback on behavior 4.1 Instruction on how to perform the behavior 4.2 Information about antecedents 5.1 Information about health consequences 10.3 Nonspecific reward
Grain foods	2.2 Feedback on behavior 4.1 Instruction on how to perform the behavior 5.1 Information about health consequences 10.3 Nonspecific reward
Vegetables and fruits	2.2 Feedback on behavior 3.1 Social support (unspecified) 4.1 Instruction on how to perform the behavior 4.2 Information about antecedents 5.1 Information about health consequences 10.3 Nonspecific reward
Plant protein foods	2.2 Feedback on behavior 4.1 Instruction on how to perform the behavior 5.1 Information about health consequences 6.1 Demonstration of behavior 10.3 Nonspecific reward
Animal protein foods	2.2 Feedback on behavior 4.1 Instruction on how to perform the behavior 5.1 Information about health consequences 10.3 Nonspecific reward

### Phase Ib: Refine

#### User Testing Session 1: Drinks and Grain Foods

The first user testing session assessing the drinks and grain foods modules occurred with student participants in grade 5 (8/12, 67% male) and grade 6 (10/18, 56% male). It was observed that grade 6 students progressed through the app at a faster rate than grade 5 students and seemed less engaged and interested in the app. Overall, 83% (25/30) agreed that the app was fun and 83% (25/30) agreed that the goals of the app were clear. Confusion and frustration in navigating the gameplay portion of the modules was also observed. Only 29% (8/30) of students agreed that the robot was easy to control. Compared with male students, female students appeared to be more interested in the healthy eating content and were more likely to want to play the app again. On the basis of this feedback, the aims for the next iteration of the app were to include more explicit tutorial instructions, improve the game controls and the movement of the robot, increase the use of gamification to enhance engagement, and clearly state the goals of the modules.

#### User Testing Session 2: Drinks

The next iteration of *Foodbot Factory* was tested with grade 4 student participants (10/20, 50% male). A direct observation revealed that students were more engaged with the dialogue and most students (14/20, 70%) demonstrated a high interest in reading the healthy eating content. Some students (4/20, 21%) showed visual frustration or confusion during game play and asked for help. Those who were confused were also observed skipping quickly through dialogue, indicating that they felt the module had too much reading. On basis of the observation, the students’ ability to move the robot improved from the previous iteration. An improvement in the clarity of the app goals and the student’s perceived enjoyment of the app was observed with this iteration of the app (Table 3). When students were asked about their favorite part of the game, 30% (6/20) identified the app characters and dialogue humor, whereas 25% (5/20) favored the *food quiz* gamified component and 15% (3/20) identified learning about nutrition. Students demonstrated learning through using the app: 90% (18/20) of students correctly identified water as the best beverage choice and responded as follows: “Water is needed to stay hydrated.”; “Water, because if you’re thirsty you can stay hydrated.” Some students provided extended commentary, stating “60% of your body is water.” and “I learned even more about having pop less.” The interviews demonstrated that students understood the key healthy eating messages embedded in the app as they recalled and explained why water is the best beverage choice for health and hydration.

**Table 3:** Summary of quantitative data for the drinks module (sessions 1-4; session 1 occurred with students in grades 5 to 6. Sessions 2 and 3 occurred with students in grade 4. Session 4 occurred with students in grades 4 to 7.)

User testing session		Strongly disagree and disagree, n (%)	Neutral, n (%)	Strongly agree and agree, n (%)
**The app was fun**				
	Session 1 (N=30)	3 (10)	2 (7)	25 (83)
	Session 2 (N=20)	1 (5)	0 (0)	18 (95)
	Session 3 (N=14)	0 (0)	1 (7)	13 (93)
	Session 4 (N=21)	0 (0)	2 (10)	19 (90)
**The goals of the app were clear**				
	Session 1	4 (13)	1 (3)	25 (83)
	Session 2	1 (5)	0 (0)	19 (95)
	Session 3	1 (7)	1 (7)	12 (86)
	Session 4	0 (0)	1 (5)	20 (95)

#### User Testing Session 3: Drinks

The next iteration of the drinks module was tested with grade 4 student participants (12/14, 86% male). Most students continued to agree or strongly agree with the statements that the app was fun and the goals of the app were clear (Table 3). The major modification to this iteration was the addition of two gamified *food catches* to introduce more gameplay, which the team hypothesized would increase engagement and interest in the app. However, more students skipped most of the dialogue compared with session 2 (Table 4). A total of 6 students demonstrated visual frustration or confusion, which was related to the *food catch,* as students had skipped through the added tutorial and did not understand what to catch to obtain points. Fewer students provided the correct answer when prompted to choose which drink is the best choice for health, with 57% (8/14) stating water. This may suggest that gamified components need to be carefully incorporated so that educational messages are not lost. Conversely, among the 36% (5/14) of students who incorrectly stated that milk was the best choice, many were able to express their knowledge of water during the interview. The high positive response of milk may reflect students’ taste preference for milk over water or baseline knowledge from parents and care providers rather than a lack of knowledge on the importance of water for health and hydration.

**Table 4:** Summary of observational data for sessions 2 to 5.

User testing session	High reading interest^a^, n (%)	Skipped most of the dialogue, n (%)
Session 2 (N=20)	14 (70)	2 (10)
Session 3 (N=14)	7 (51)	2 (14)
Session 4 (N=21)	13 (60)	2 (10)
Session 5 (N=17)	13 (75)	2 (13)

^a^High reading interest was defined as an observational rating of 4 or 5 on a 5-point Likert scale assessing engagement with dialogue content. Skipping dialogue was assessed via an observational note with options of none, some, and most. Students who skipped most of the dialogue were observed flipping quickly through the dialogue and not reading the majority of the content.

#### User Testing Session 4: Drinks and Grain Foods

The next testing session occurred with summer camp student participants at Ontario Tech University (14/21, 66% male; grades 4-6). The drinks module was unmodified from session 3. The grain foods module was modified to include a new interactive component to teach about different parts of the grain kernel and play an additional *food sort* minigame to increase the variety between the gamified portions of each module. It was hypothesized that engagement would increase by including a variety of interactive components. Observations during gameplay were similar to previous sessions; however, some students appeared to disengage, ie, appearing bored, after playing more than one module. This may explain why observational data showed a slight decline in engagement compared with sessions 2 and 3 where only one app module was tested (Table 4). As in previous sessions, students connected with the game characters and humorous dialogue, with 2 students commenting the following: “That was really fun!” In this testing session, no students showed visual frustration or confusion, potentially indicating that the usability of the game and clarity of instructions had improved.

Knowledge gained by using *Foodbot Factory* improved from previous sessions. For the drinks module, all students (21/21) replied that water is the best for quenching thirst, with students recognizing that “You need it [water] to survive and stay hydrated” and “60% of the body is made of water.” Students also conveyed their knowledge of milk and sugary drinks. Students’ comments included the following: “Milk has calcium and Vitamin D and makes bones grow bigger and stronger” and “Pop is a treat like chocolate. You get cavities from the sugar.” For the grain foods module, 70% (15/21) of students recalled that whole grain bread has more fiber than white bread, with students stating “Whole grain bread has more fiber and is better for you than white bread because of fiber” and “White bread has less fiber because they remove the bran and the germ.” On average, students were able to fulfill the learning objectives for both the drinks and grain foods modules. Importantly, there were considerable improvements on two key metrics of concern. Overall, 90% (19/21) of students agreed with the statement “the app was fun” and 95% (20/21) agreed “the goals of the app were clear” (Table 3)*.* Although observational data suggest that students may disengage when playing multiple modules in one sitting, the self-reported questionnaire data confirm that students found the game engaging and usable.

#### User Testing Session 5: Vegetables and Fruits and Protein Foods

In this last user testing session among grade 4 student participants (9/17, 55% male), the first iterations of the vegetables and fruits module or the protein foods module were tested. On the basis of the iterative feedback and lessons learned from previous testing sessions, these modules included multiple interactive components. For example, interactive components allowed students to choose the vegetables and fruits to learn about or to cook pasta, and there was added variation between the *food sort* and *food catch* minigames.

Similarly, high levels of engagement as seen in previous user testing sessions were observed (Table 4). Among those who played the vegetables and fruits module (N=8), 75% (6/8) of students recalled a key healthy eating message, with one student stating “They [vegetables & fruits] have lots of vitamins and minerals” and another stating “Most of the stuff [nutrients] helps your body and when you eat it, it helps your body work.” By contrast, only 55% (5/9) of students who played the protein foods module (N=9) were able to recall a key healthy eating message. The team concluded that this first iteration of the protein foods module had an ambitious number of key messages and learning outcomes. Therefore, the protein foods module was divided into animal protein foods and plant protein foods to enhance the focus and clarity of healthy eating messages and improve students’ ability to meet the learning objectives. Overall, the majority of users still found the modules to be fun, with clear goals, easy to use, and easy to read (Table 5). A total of 71% (12/17) of students agreed with the statement “I want to keep playing,” indicating that *Foodbot Factory* is an engaging and acceptable way for students to learn about food, nutrition, and healthy eating (Table 5).

**Table 5:** Summary of quantitative data for user testing session 5 (N=17)

Question	Strongly disagree and disagree, n (%)	Neutral, n (%)	Strongly agree and agree, n (%)
The goals of the app are clear	1 (6)	0 (0)	16 (94)
The app was easy to use	1 (6)	4 (24)	12 (71)
The words in the app are easy to read	1 (6)	4 (24)	12 (71)
I want to keep playing	0 (0)	5 (29)	12 (71)

#### Subgroup Analyses

Differences in engagement between male and female student participants were observed from session 1, and it was hypothesized that female students were more engaged with the app than male students. The subgroup analysis (52/87, 60% male) indicated that female students were significantly more likely than male students to agree that they wanted to keep playing (29/35, 83% vs 37/52, 71%; *P*=.03). Trends indicate that female students tended to agree that the app was fun (33/35, 97% vs 45/52, 86%; *P*=.11) and tended to disagree that they were bored (28/35, 82% vs 39/52, 75%; *P*=.09) compared with male students; however, these differences were not statistically significant.

To address the differences in engagement between male and female students, the development team made significant efforts to enhance overall engagement with each iteration of *Foodbot Factory* by further incorporating games and humor as a strategy to better appeal to male students. A voiceover was also added to the app to enhance overall engagement and make the content more accessible for those who may have difficulty reading. Although female students tended to show higher levels of engagement compared with male students, an overall high proportion of male students still found the app fun, and they demonstrated an interest and ability to learn through gameplay, as evidenced by qualitative feedback and observation.

## Discussion

### Principal Findings

The iterative development and evaluation of *Foodbot Factory* has led to the creation of an evidence-based and engaging mHealth app to help Canadian children learn about healthy eating and nutrition. The interdisciplinary team of experts in nutrition, education, and game design, in collaboration with policymakers (Health Canada), ensured that the app is consistent with the latest nutrition recommendations, incorporated current advances in gaming and education technology, and can be easily implemented in the classroom. The multimethod user testing data demonstrate that the majority of *Foodbot Factory’s* target audience find the app engaging and usable, providing evidence that this public health intervention can help Canadian children learn about nutrition. *Foodbot Factory* has undergone evaluation consistent with *Phase I: Design* in the ORBIT model and is currently being evaluated in a randomized controlled trial as a part of *Phase 2: Preliminary Evaluation* [19]. A strength of this research is the use of an interdisciplinary team of experts to facilitate and participate in development. An interdisciplinary approach is not always implemented when designing electronic health and mHealth interventions [48], but it should be encouraged to ensure that the multiple disciplines involved in developing high-quality digital interventions are represented throughout the development process. Our research and development team worked effectively in a collaborative fashion through weekly scheduled meetings until the app development was fully complete. We further leveraged our strong relationships with partners across disciplines, including governments, nongovernmental organizations, and school boards, to obtain diverse expert opinions and conduct the research presented in this study.

### Strengths and Limitations

In comparison with similar studies that have conducted iterative user testing methodologies to develop mHealth interventions, the research presented here has implemented significantly more iterative testing sessions with the target audience. The majority of other app development studies that report user testing often implement various stepwise iterative processes throughout development; however, many only utilize one testing session with their target audience [49-53]. The use of multiple testing sessions for *Foodbot Factory* is a strength of this study as it allowed the research team to improve engagement and usability of the app. Multiple testing sessions also enabled app evaluation with a larger sample size and tailored the content of the app to the diverse needs of *Foodbot Factory’s* target audience. Finally, other studies that report on the development of mHealth interventions have not implemented multiple methods in their user testing sessions [51,53]. The use of multiple methods in this study provided the research team with a set of rich and comprehensive data on the user experience, increasing the efficiency and effectiveness of each successive iteration of *Foodbot Factory*. The development of future mHealth apps and interventions would be best informed by iterative testing to ensure that changes implemented to each iteration meet the needs of the target audience and advance the goals of the intervention. Testing should also utilize mixed method data collection, as recommended by the Iterative Convergent Design for Mobile Health Usability Testing [23].

Although there were positive outcomes to this project, we acknowledge that there were limitations. Observation was relied on as a way to assess user engagement with app dialogue content and the rate of progression; however, these measures of user engagement could have been enhanced by collecting quantitative interapp analytic data. Factors that influence the dietary intakes of children are often beyond the control of a young child (eg, socioeconomic status, parental influence, and neighborhood food environment) [54]. However, the knowledge gained from playing *Foodbot Factory* can assist children in making healthier dietary decisions where possible, and the foundational knowledge may stay with the child over the long term. Testing results suggest that student participants may disengage after playing more than one module during a session and that female students may be more engaged than male students. However, these findings relay important information for implementation into the classroom setting, suggesting that learning will be optimized by using the app for short periods over several days. *Foodbot Factory* can be played in a flexible format, providing users with a choice in the number of modules played in a given session. The app has also been designed with multiple gamified components that vary through each module to appeal to a variety of learning and gameplay preferences. Future research with *Foodbot Factory* includes a randomized trial measuring its impact on nutrition knowledge and development of a knowledge translation strategy to ensure maximal reach and adoption among teachers, health professionals, and parents.

In summary, *Foodbot Factory* is a viable and an engaging app that has the potential to improve the nutrition knowledge of Canadian children. The app aligns with the latest dietary recommendations for Canadians and curriculum expectations for most Canadian provinces [40,45]. *Foodbot Factory* utilizes increasingly available technologies, providing a contemporary means for children to learn about nutrition at home and in the classroom. The knowledge gained during childhood may be sustained into the adolescent years, with the intended benefit of improving dietary intake and reducing the risk of chronic diseases associated with poor diet quality.

## Appendix

Multimedia Appendix 1 Summary of the Foodbot Factory content.

Multimedia Appendix 2 A Foodbot Factory gameplay video.

Multimedia Appendix 3 Foodbot Factory gamification characteristics.

## Abbreviations

BCT: behavior change technique
CFG: Canada’s Food Guide
mHealth: mobile health
ORBIT: Obesity-Related Behavioral Intervention Trials
