# Supplementary material for: Optimizing Child Nutrition Education With the Foodbot Factory Mobile Health App: Formative Evaluation and Analysis
Source: JMIR Form Res. 2020 Apr 17;4(4):e15534. doi: 10.2196/15534 (PMC7195667; doi:10.2196/15534)
Supplement: Multimedia Appendix 1 [file formative_v4i4e15534_app1.docx]

**Multimedia Appendix 2: Foodbot Factory Game Build**

Introduction and Menu


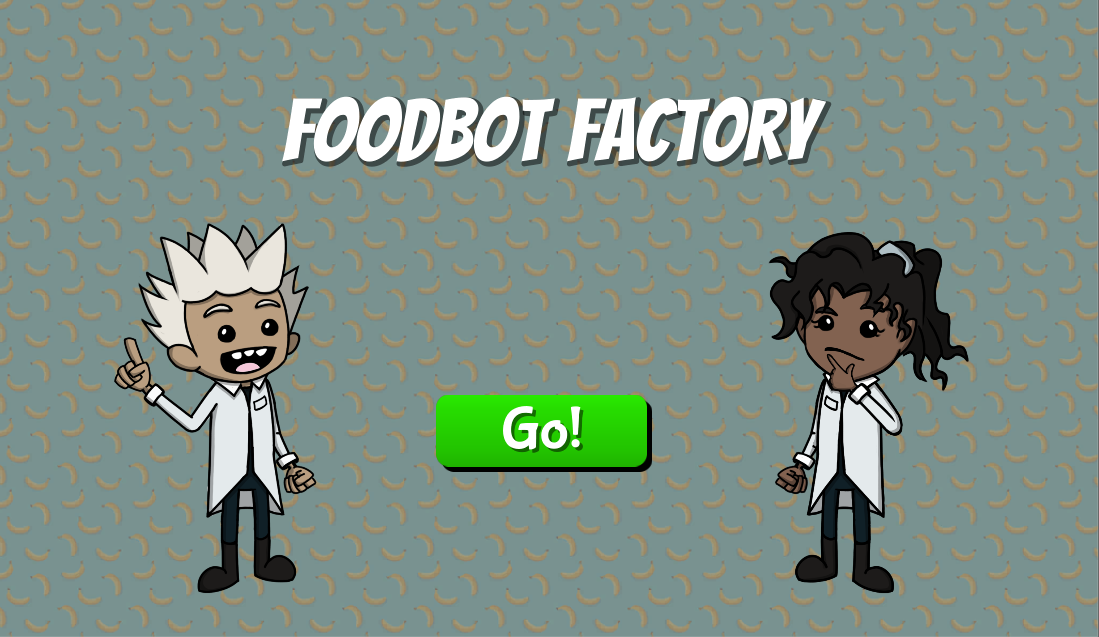

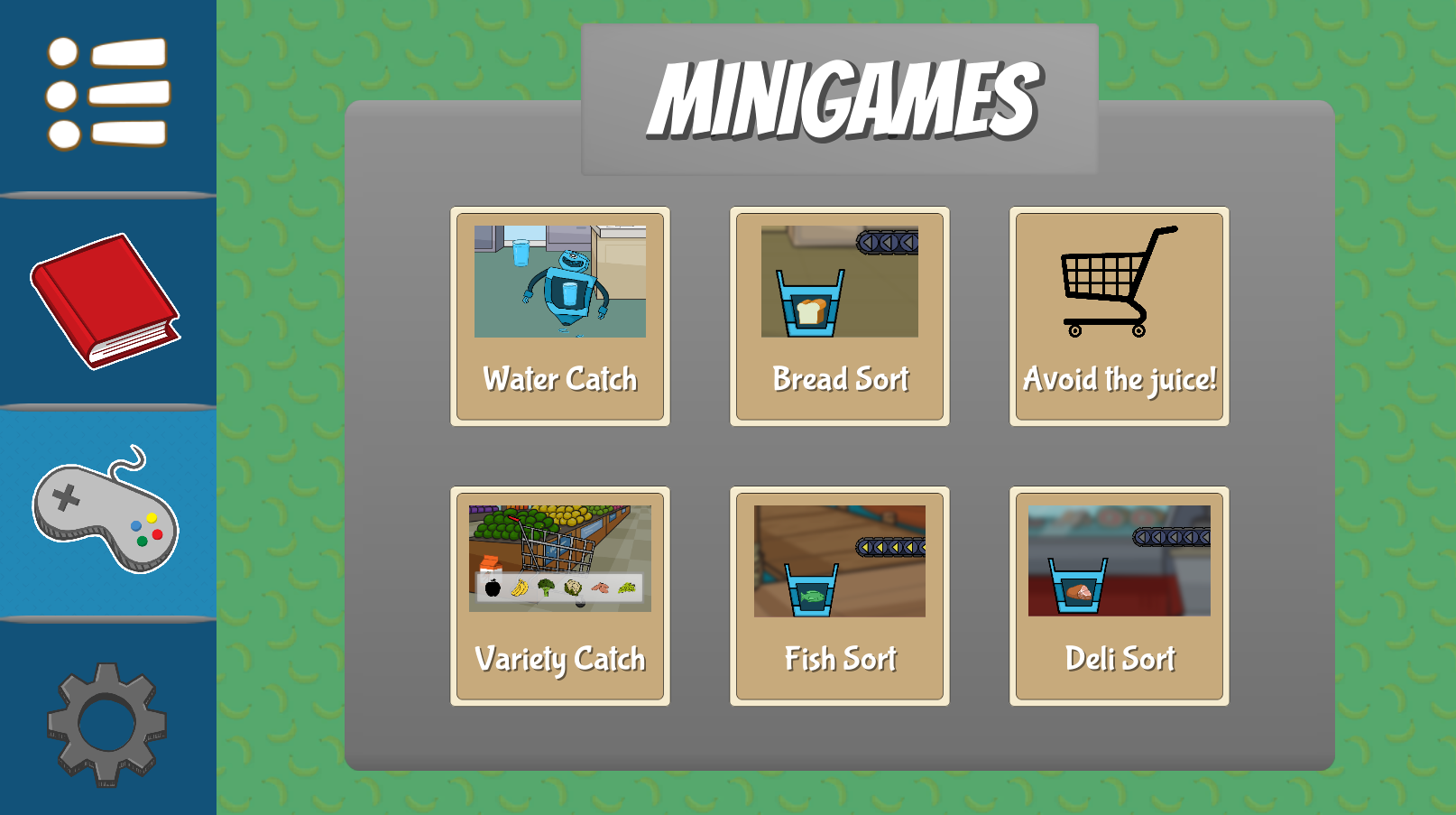


Start Screen Mini Game Selection


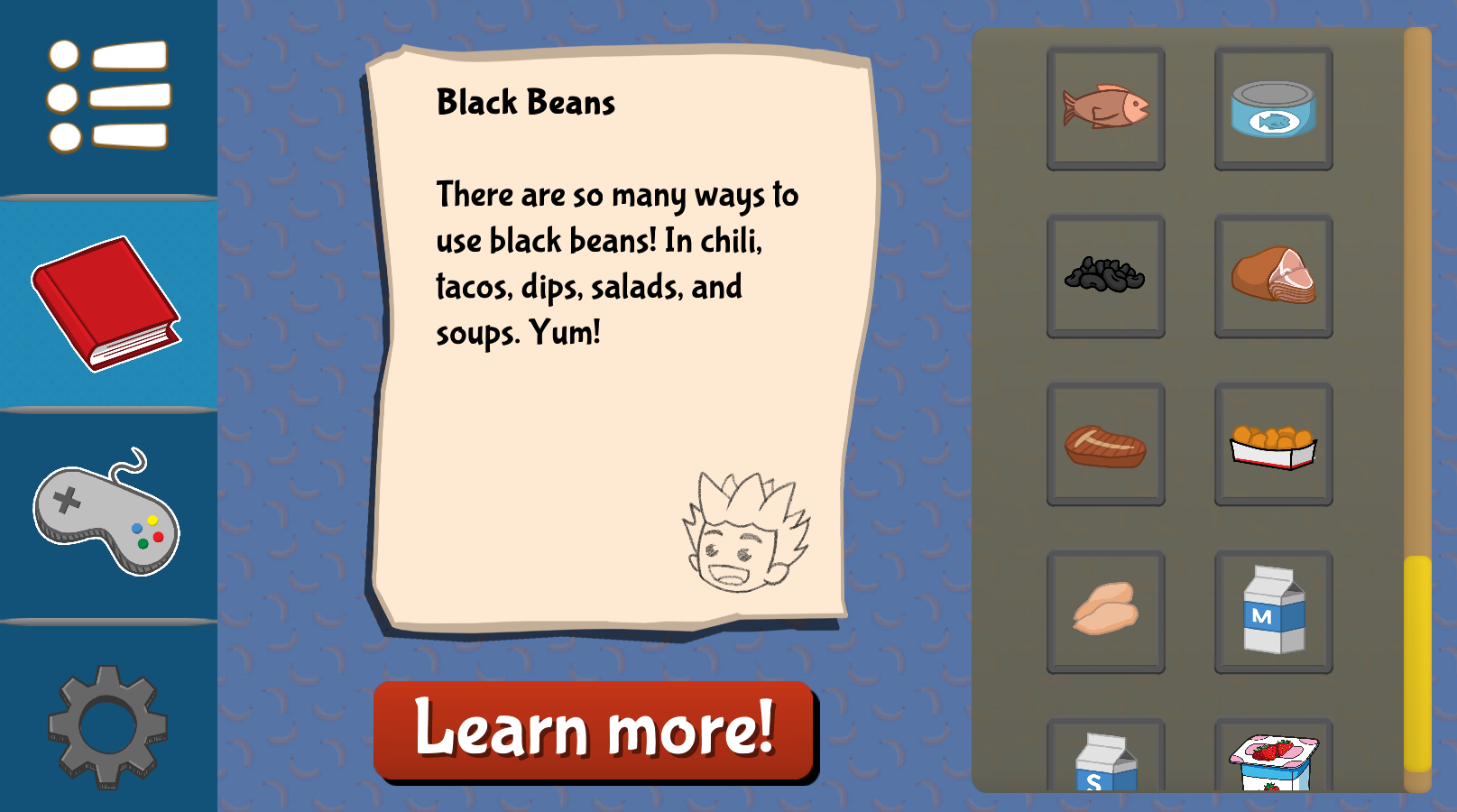

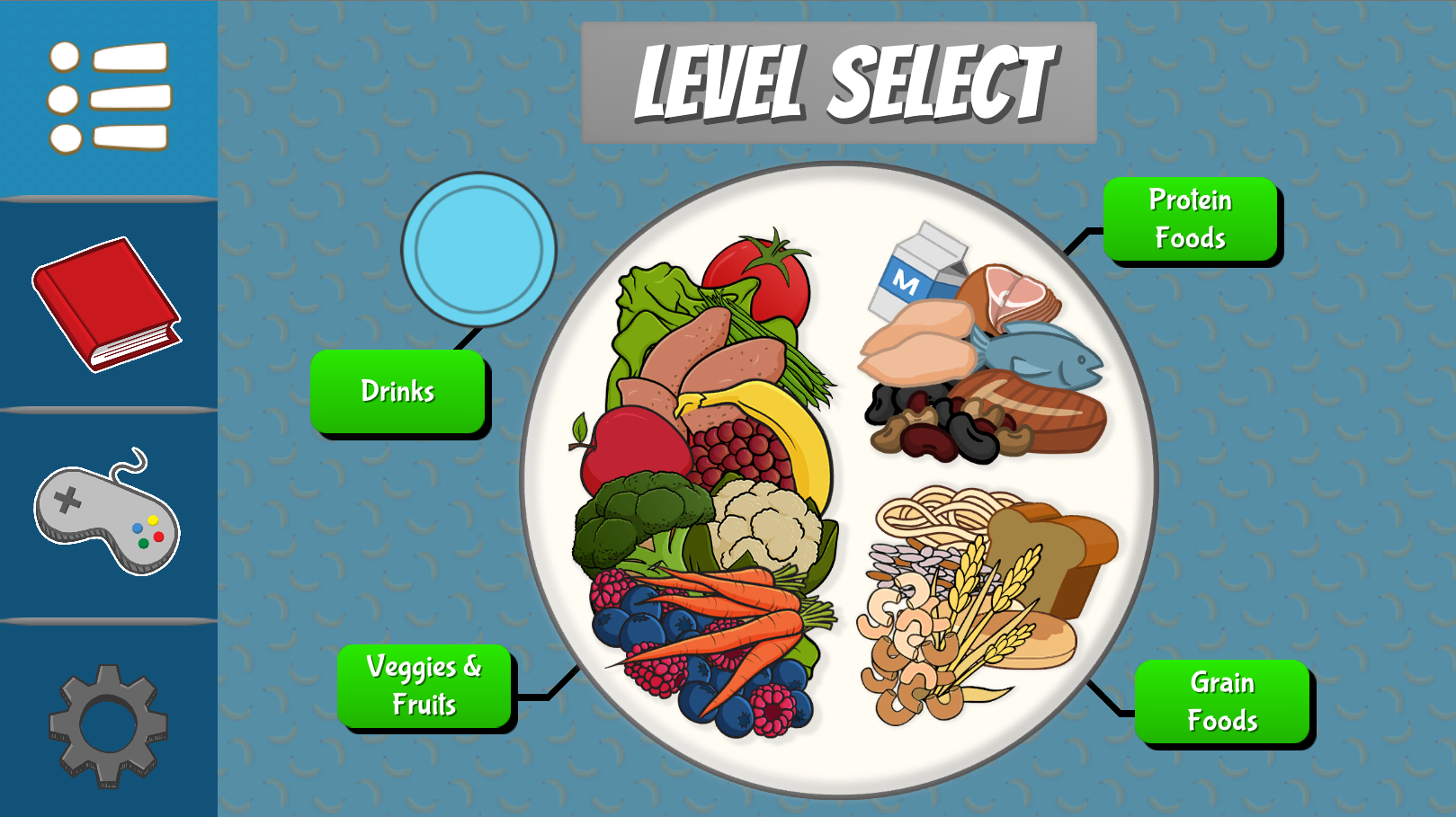


Food Log Module Selection Menu

Module 1: Drinks

**Learning Objectives**

1. Recall the best beverage choice for staying hydrated
2. Describe the health effects of different types of beverages
3. Recall different types of sugary drinks

**CFG Messages Included:**

- Water is the best choice to stay healthy and hydrated.
- Make water your drink of choice instead of sugary drinks.
- Sugary drinks can cause cavities, diabetes and obesity.
- Low-fat dairy and unsweetened soy milk are healthy options.

**User Flow Diagram**

**Sample Screenshots**


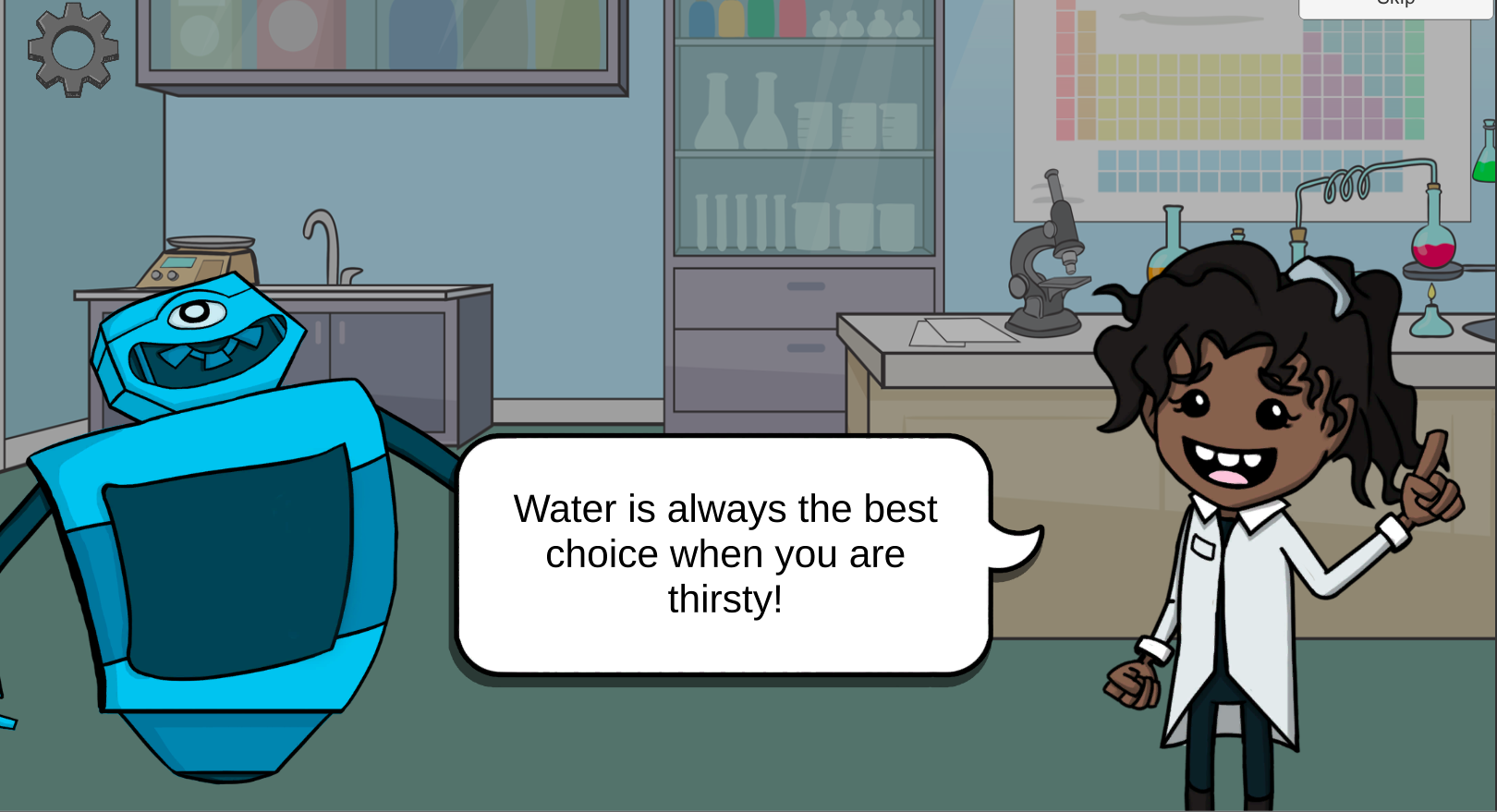

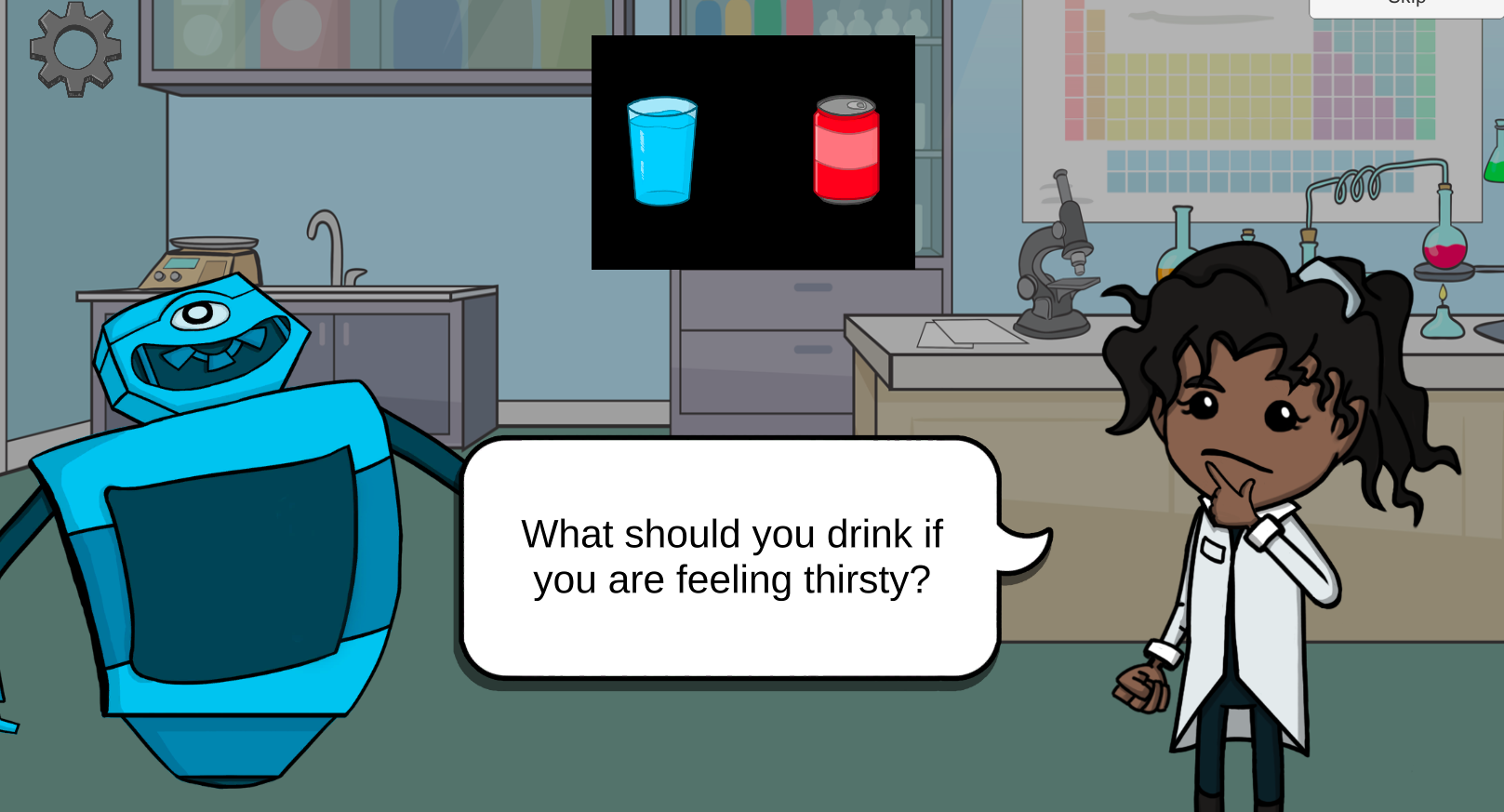


Sample Dialogue Drinks Quiz

**
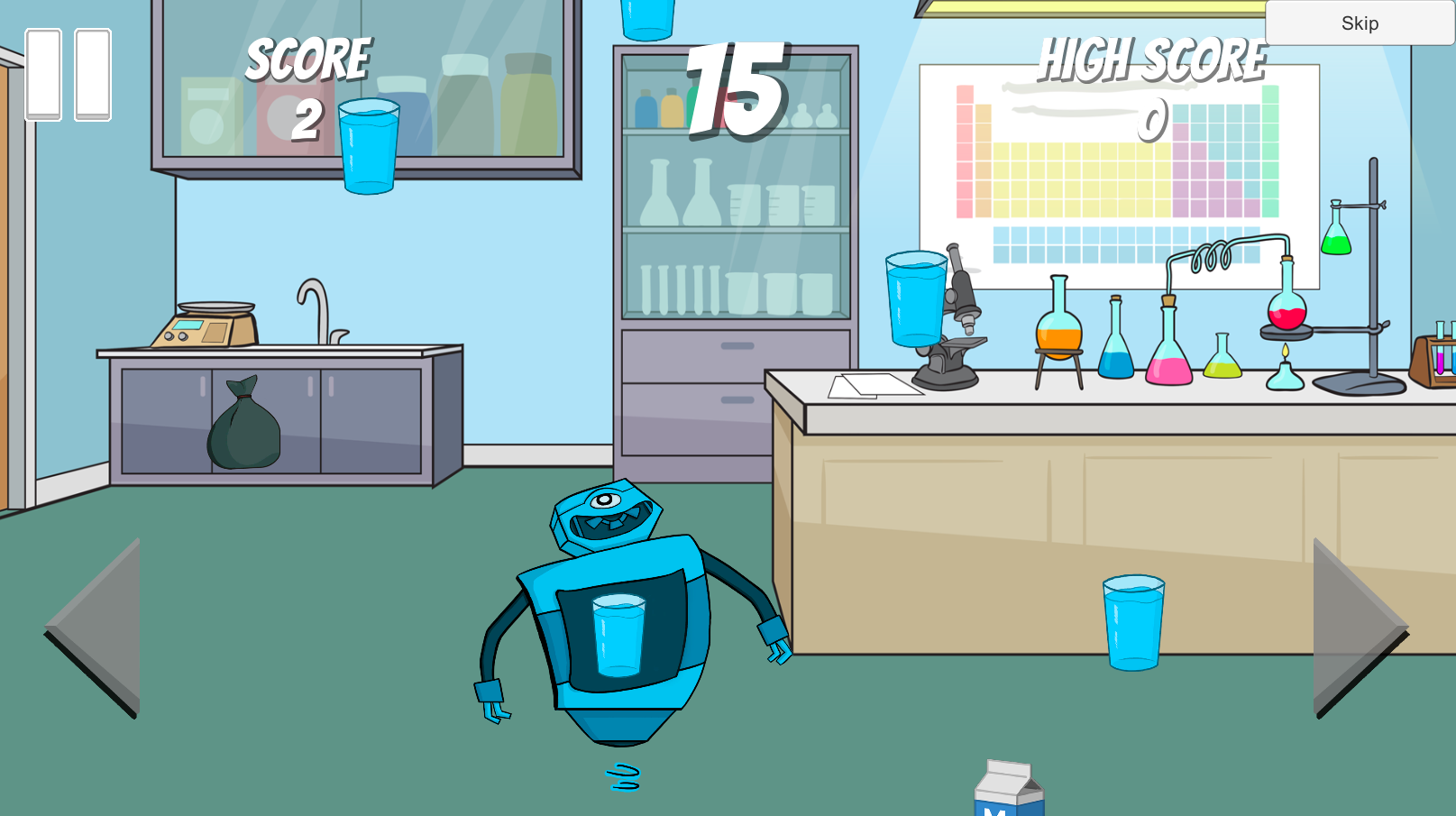
**

Drinks “Food Drop” game

Module 2: Grain Foods

**Learning Objectives**

1. Explain the nutritional differences between whole grain and refined grain products
2. Recall the components of the grain kernel and how grains are refined
3. Describe why consuming fibre is important for health

**CFG Messages Included**

- Choose whole grain foods more often than refined grain foods
- Whole grain foods contain more fibre, vitamins, and minerals than refined grain foods
- Fibre helps you feel full, keeps your digestive system healthy and prevents heart disease and cancer

**User Flow Diagram**

**Screenshots**


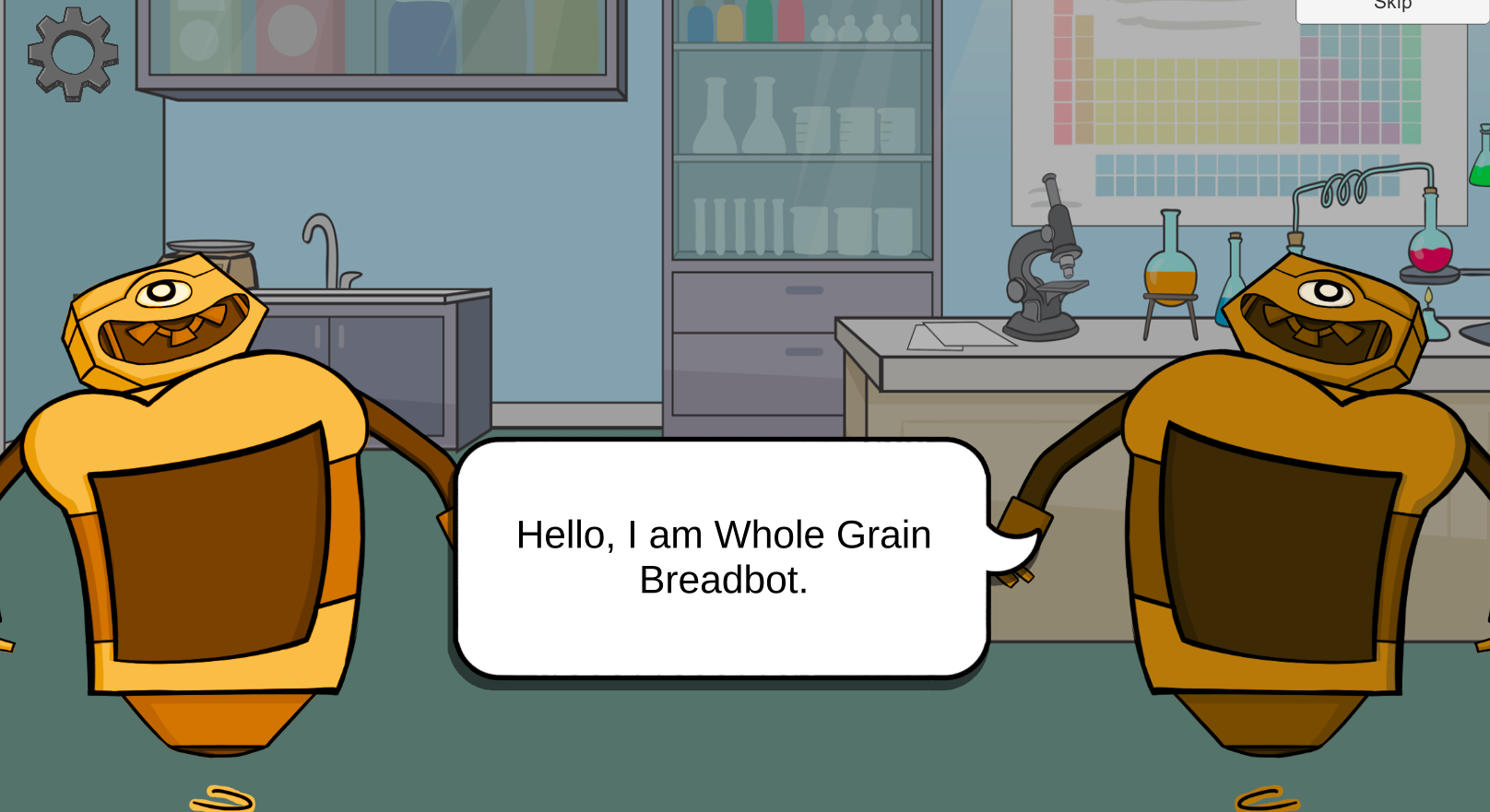

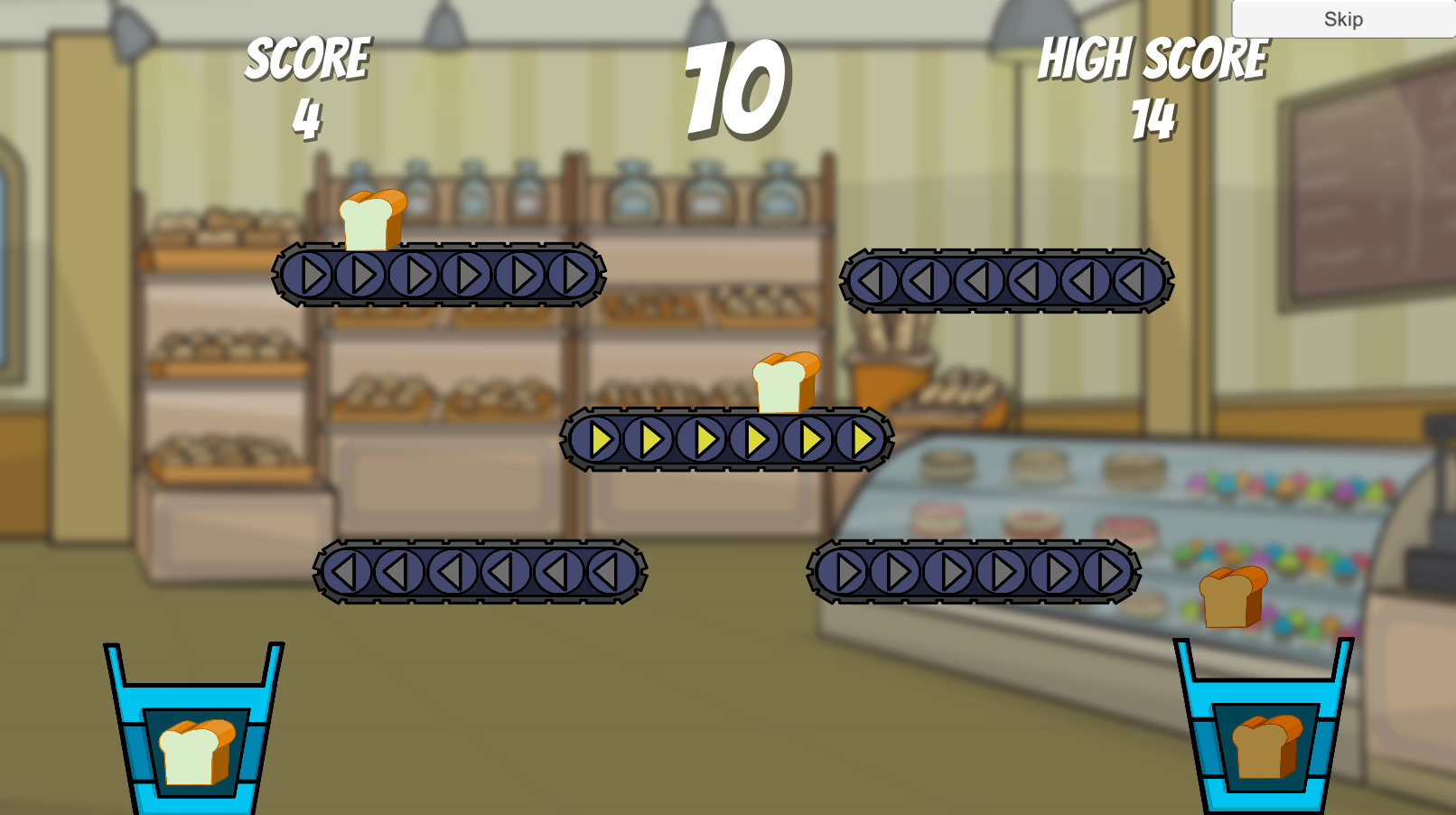


Sample Dialogue Refined & Whole Grain “Food Sort”


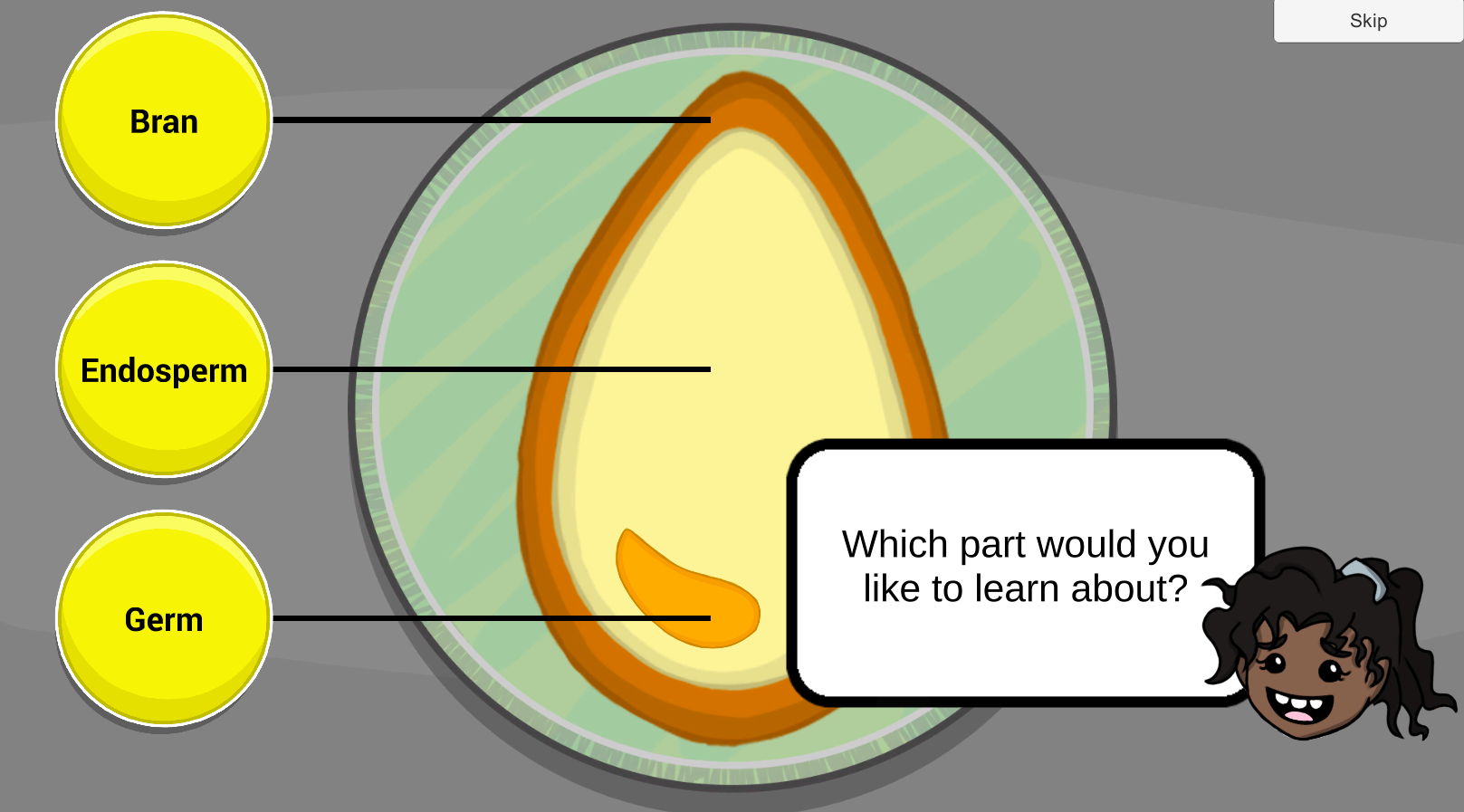

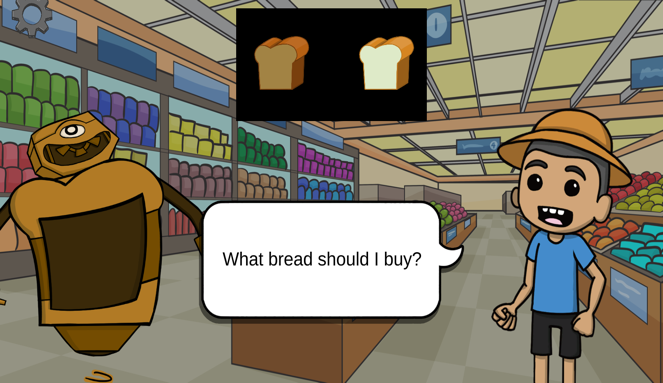


Interaction: Grain Kernel Grain Foods Quiz

Module 3: Veggies and Fruit

**Learning Objectives**

1. Recall the amount of vegetables and fruits that should be consumed with a meal
2. Explain why vegetables and fruits are a healthy choice
3. Describe which forms of vegetables and fruit are healthiest to consume (i.e., canned, frozen, juice).

**CFG Messages Included**

- ½ of your plate should consist of vegetables and fruits
- Vegetables and fruits are a good source of fibre, vitamins, and minerals
- Consume a variety of vegetables and fruits daily that are different colours, textures, and shapes
- Canned vegetables and fruits may contain added sodium and/or sugar but are still healthy choices
- Whole vegetables and fruits are a healthier choice than juices because they contain less sugar and more fibre

**User Flow Diagram**

**Screenshots**


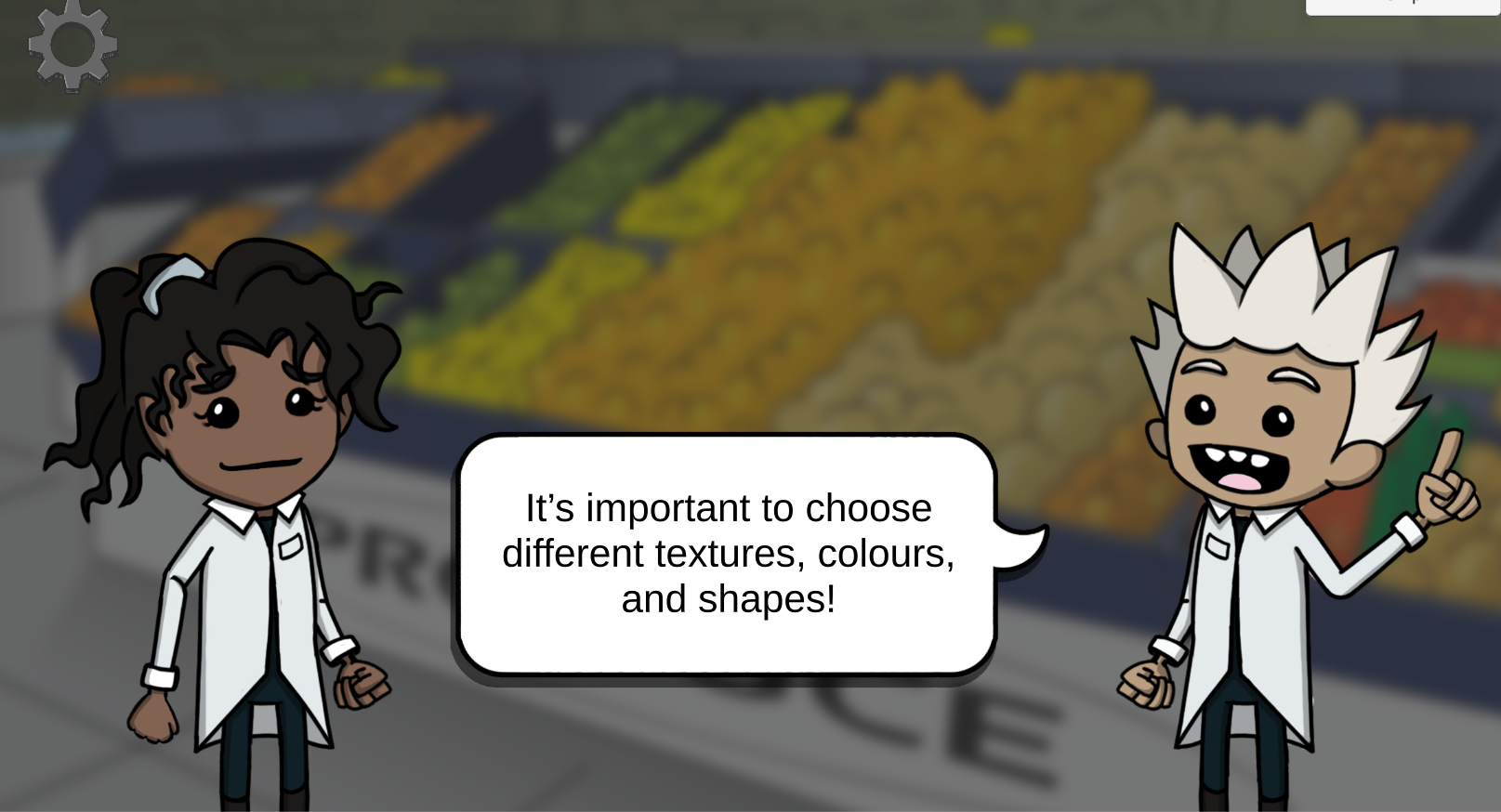

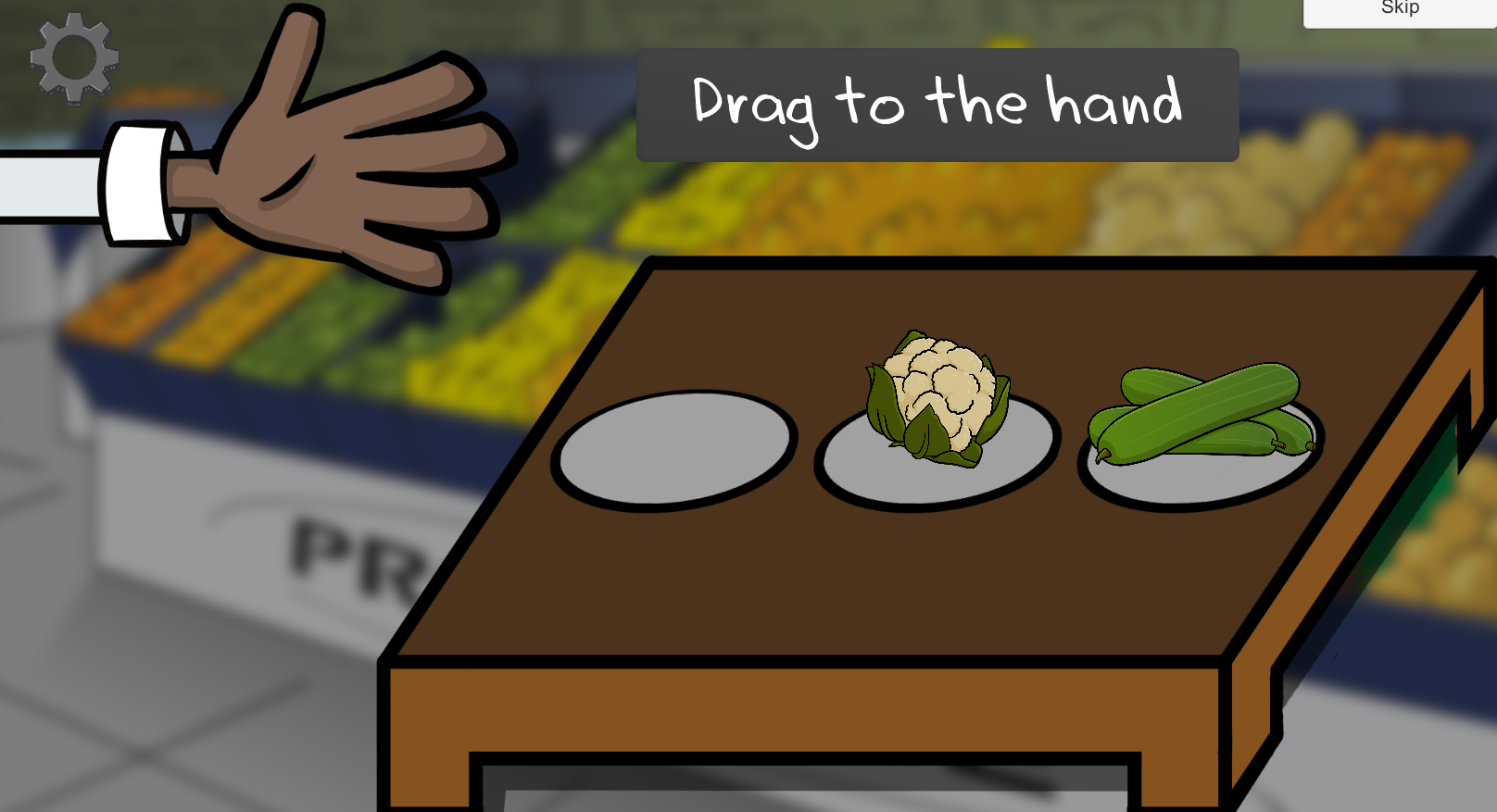


Sample Dialogue Interaction: Variety


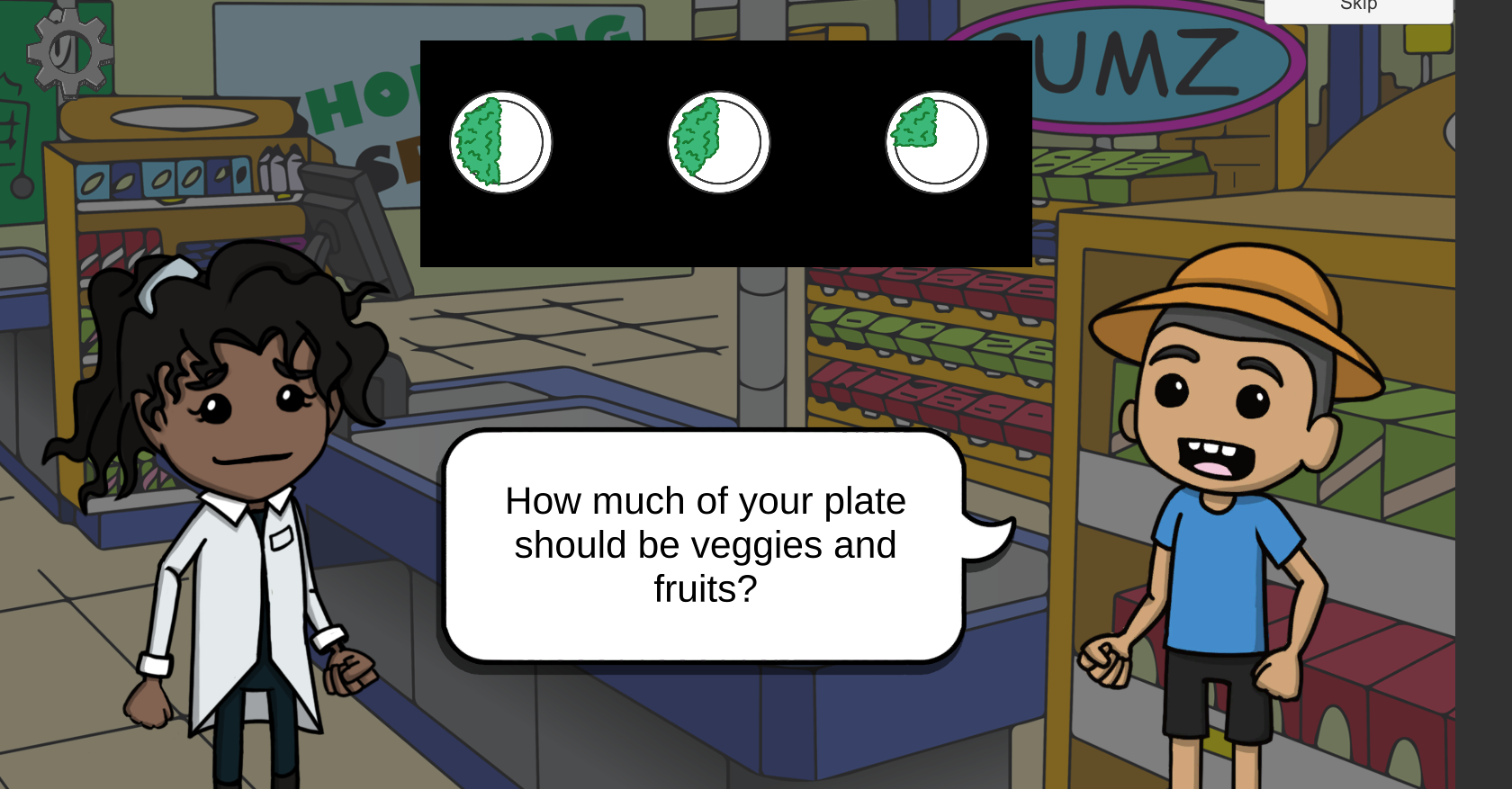

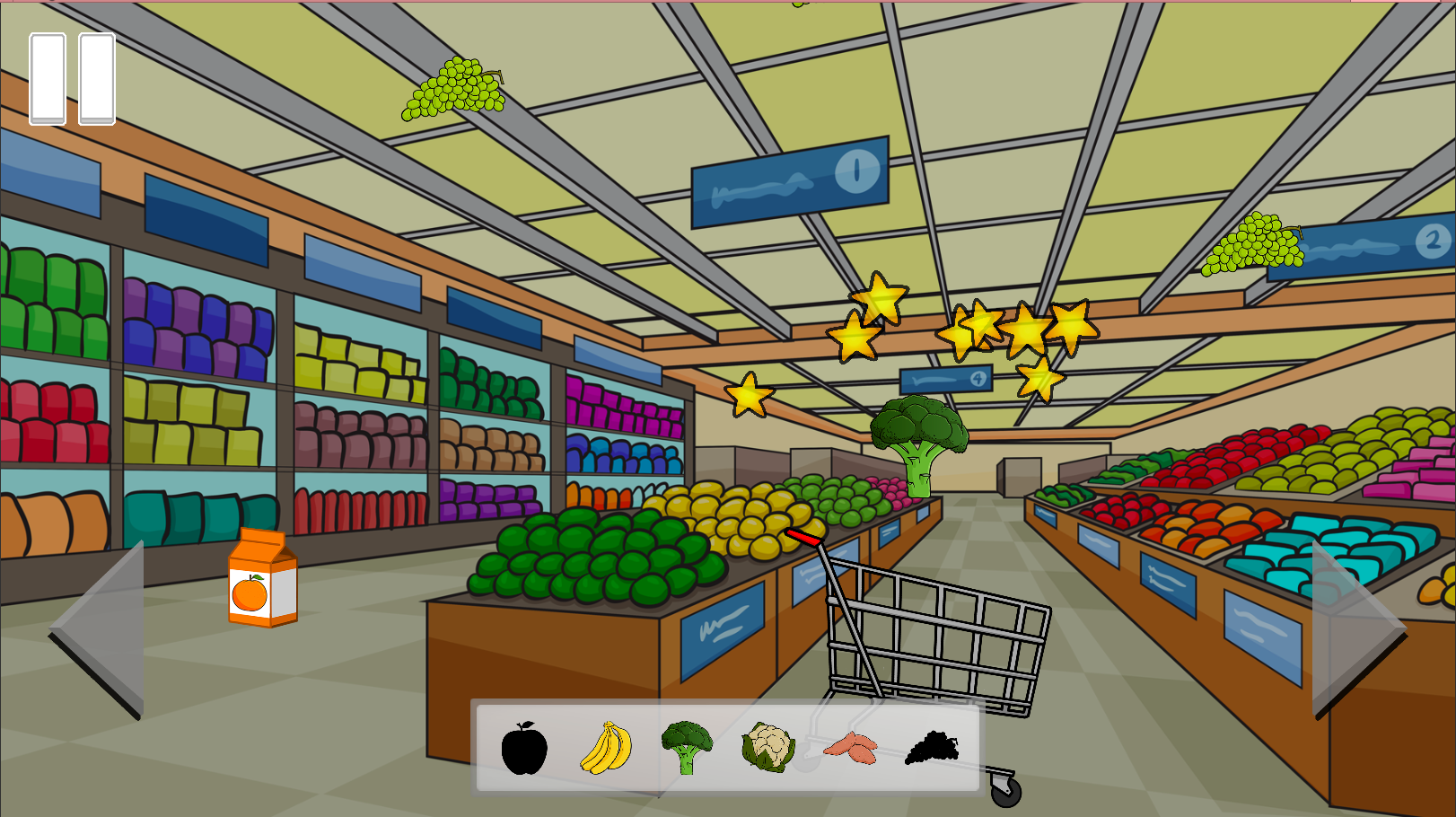


Veggies and Fruit Food Quiz Veggies and Fruit Variety “Food Drop”

Module 4: Plant Protein Foods

**Learning Objectives**

1. Describe what foods are plant-protein foods, and why they are a healthy choice
2. Recall that plant-protein foods contain fibre

**CFG Messages Included**

- Choose plant proteins most often
- Plant proteins are the only protein source that also contains fibre

**User Flow Diagram**

**Screenshots**


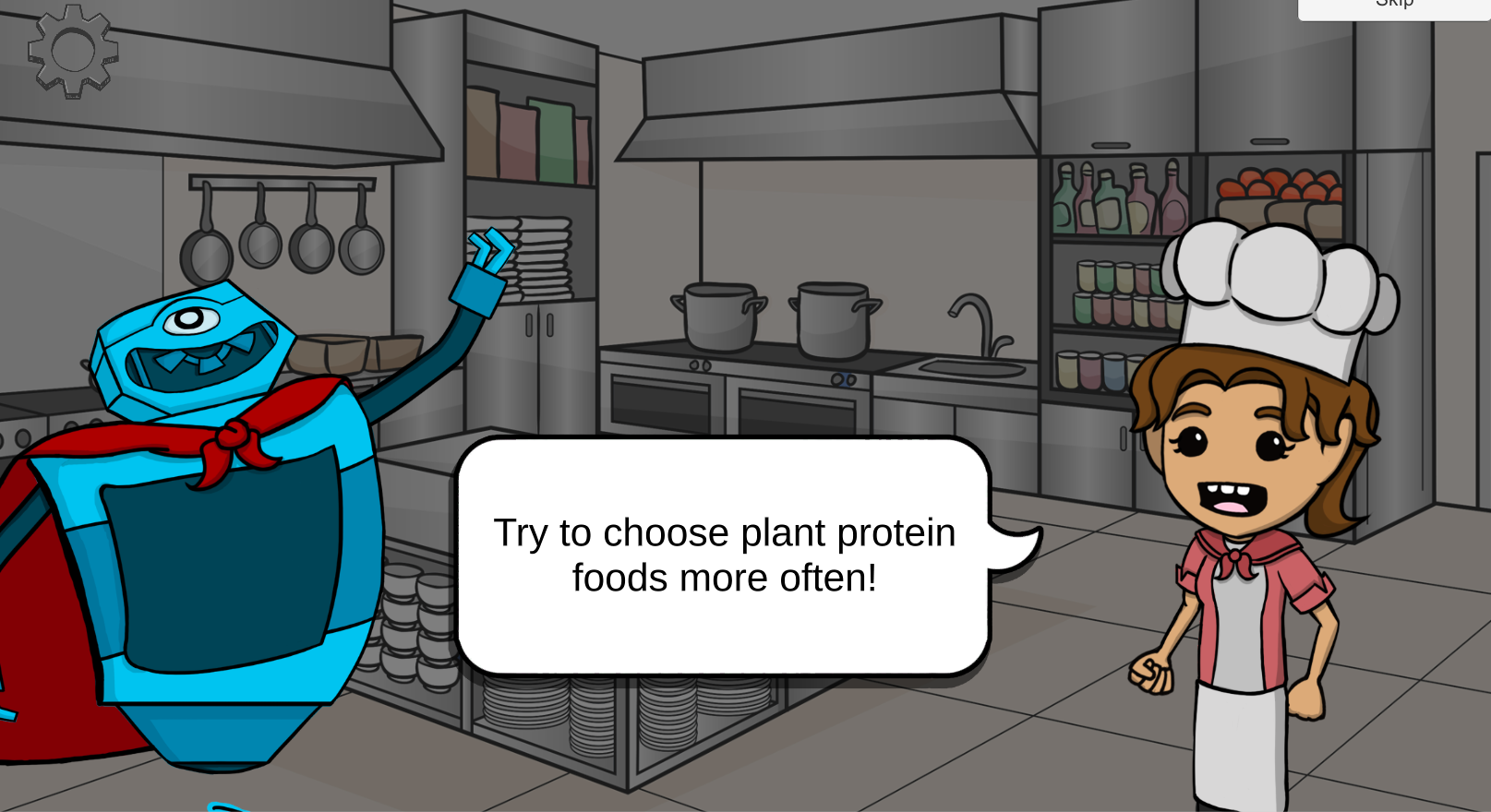

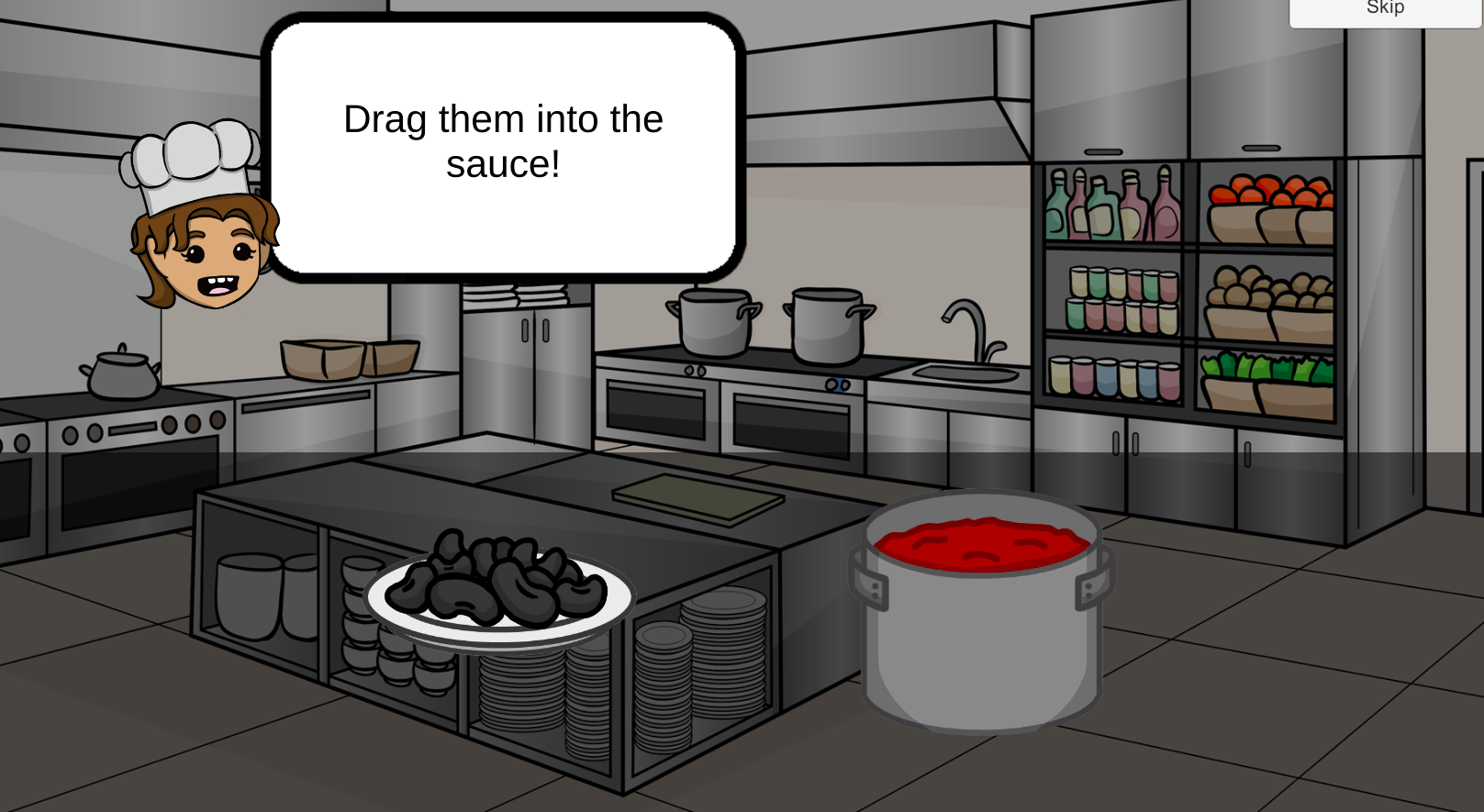


Sample Dialogue Interaction: Cooking with Plant Protein Foods


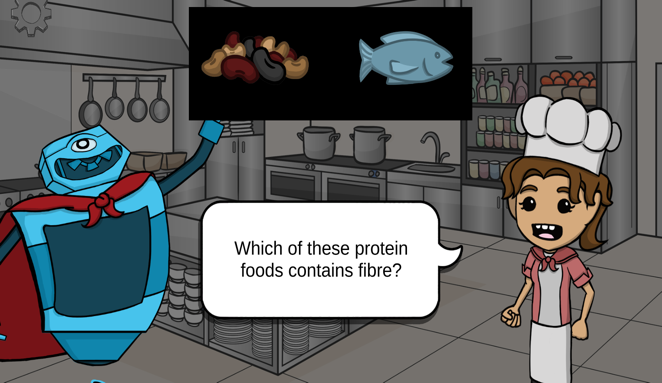


Plant Protein Foods Quiz

Module 5: Animal Protein Foods

**Learning Objectives**

1. Recall that some fats are healthy (unsaturated fats) and unhealthy (saturated fats)
2. Describe the health effects of excess dietary saturated fat and sodium.
3. Explain why processed meats should be consumed less often
4. Describe why fish are a healthy choice

**CFG Messages Included**

- Processed meats are high in sodium and less healthy saturated fats and should only be eaten occasionally and in smaller portions
- High sodium consumption can lead to health consequences like heart disease
- Fish are a good source of protein and healthy unsaturated fat

**User Flow Diagram**

**Screenshots**


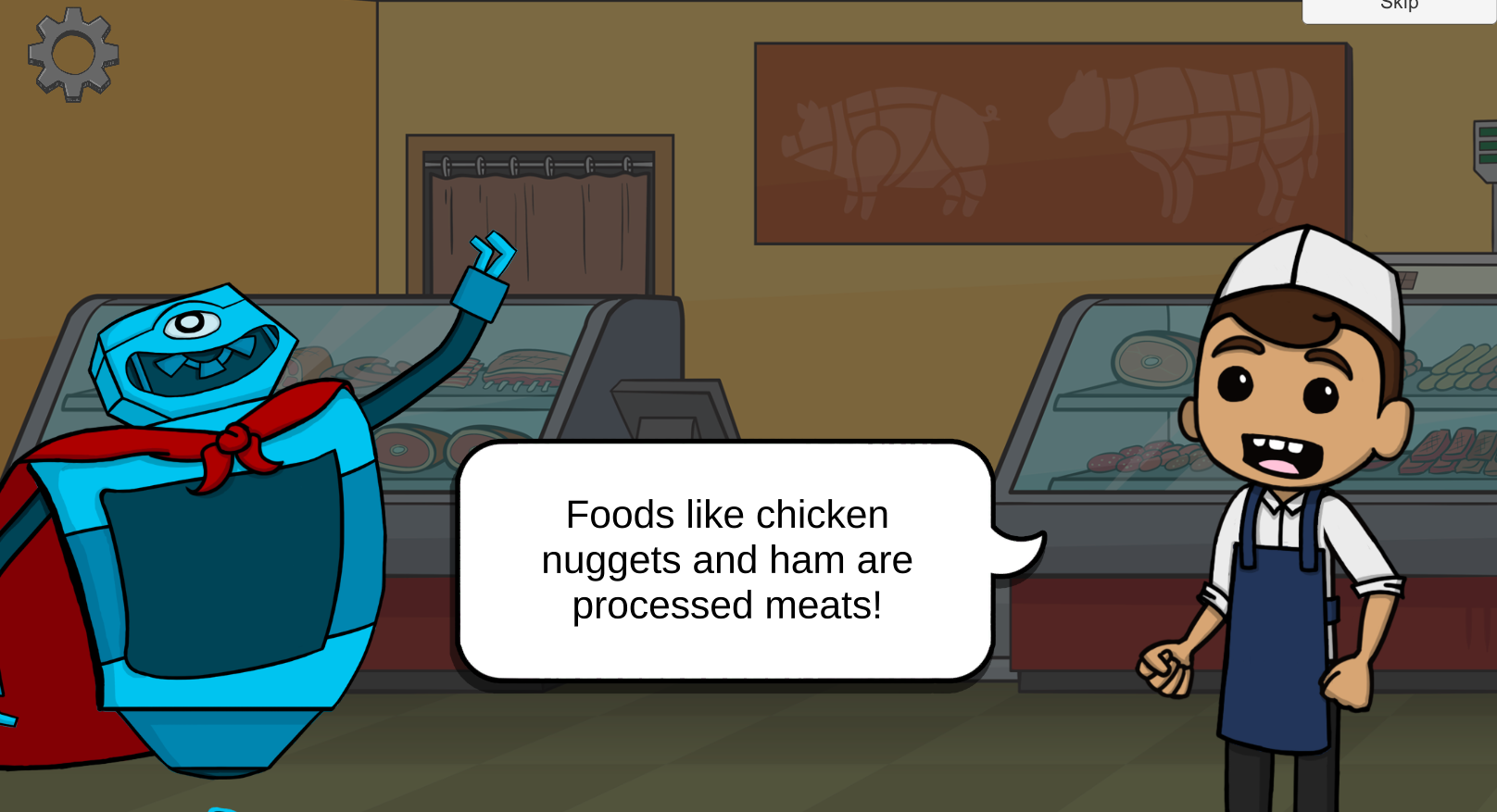

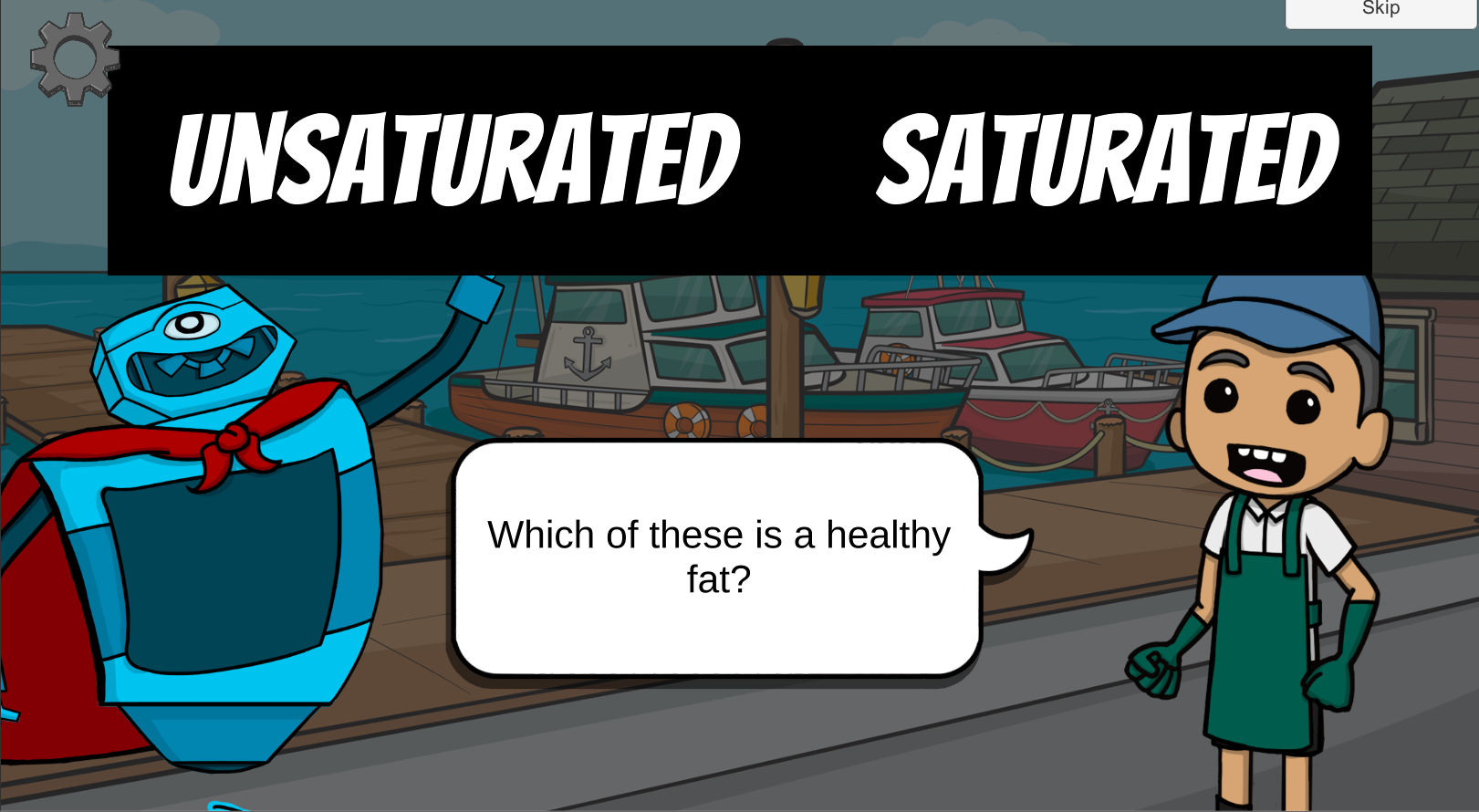


Sample Dialogue Food Quiz


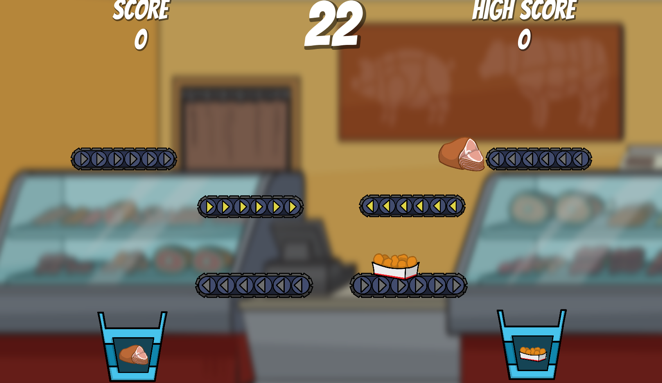

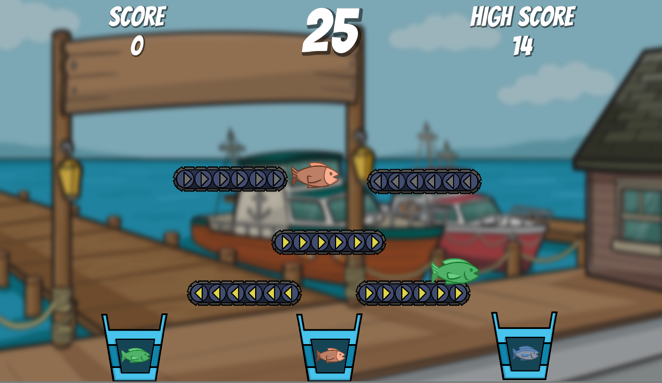


Processed Meat “Food Sort” Fish “Food Sort”
