# Supplementary material for: Optimizing Child Nutrition Education With the Foodbot Factory Mobile Health App: Formative Evaluation and Analysis
Source: JMIR Form Res. 2020 Apr 17;4(4):e15534. doi: 10.2196/15534 (PMC7195667; doi:10.2196/15534)
Supplement: Multimedia Appendix 3 [file formative_v4i4e15534_app3.docx]

**Foodbot Factory Gamification Characteristics**

| Dimension ^a^ | Characteristics | | | |
| --- | --- | --- | --- | --- |
| Gamification concept-to-user communication | Direct | | Mediated | |
| User identity | Virtual character | Self-selected | | No |
| Rewards | Internal | Internal and external | | No |
| Competition | Direct | Indirect | | No |
| Target Group | Patients | Healthy individuals | | Health professionals |
| Collaboration | Cooperative | Supportive only | | No |
| Goal-Setting | Self-set | Externally-set | | No |
| Narrative | Continuous | | Episodical | |
| Reinforcement | Positive | | Positive-negative | |
| Level of integration | Independent | | Inherent | |
| Persuasive intent | Compliance change | Behavior change | | Attitude change |
| User advancement | Presentation only | Progressive | | No |

^a^ Since *Foodbot Factory* is focused on education, rather than behaviour change like most mHealth apps, the Taxonomy of Gamification Concepts for Health Apps [47] was modified to include a “No” option for the characteristics of user identity and goal-setting.
